# Interactive effects of pain and arousal state on heart rate and cortical activity in the mouse anterior cingulate and somatosensory cortices

**DOI:** 10.1016/j.ynpai.2024.100157

**Published:** 2024-04-25

**Authors:** Raquel Adaia Sandoval Ortega, Margot Renard, Michael X. Cohen, Thomas Nevian

**Affiliations:** a Neuronal Plasticity Group, Department of Physiology, University of Bern, Bühlplatz 5, 3012 Bern, Switzerland; b Synchronization in Neural Systems Lab, Donders Centre for Medical Neuroscience, Radboud University Medical Center, Houtlaan 4, 6525 XZ Nijmegen, the Netherlands

**Keywords:** Pain, Nociception, Sleep, Sensory processing, Anterior cingulate cortex, Somatosensory cortex, Oscillations

## Abstract

Sensory disconnection is a hallmark of sleep, yet the cortex retains some ability to process sensory information. Acute noxious stimulation during sleep increases the heart rate and the likelihood of awakening, indicating that certain mechanisms for pain sensing and processing remain active. However, processing of somatosensory information, including pain, during sleep remains underexplored. To assess somatosensation in natural sleep, we simultaneously recorded heart rate and local field potentials in the anterior cingulate (ACC) and somatosensory (S1) cortices of naïve, adult male mice, while applying noxious and non-noxious stimuli to their hind paws throughout their sleep-wake cycle. Noxious stimuli evoked stronger heart rate increases in both wake and non-rapid eye movement sleep (NREMS), and resulted in larger awakening probability in NREMS, as compared to non-noxious stimulation, suggesting differential processing of noxious and non-noxious information during sleep. Somatosensory information differentially reached S1 and ACC in sleep, eliciting complex transient and sustained responses in the delta, alpha, and gamma frequency bands as well as somatosensory evoked potentials. These dynamics depended on sleep state, the behavioral response to the stimulation and stimulation intensity (non-noxious vs. noxious). Furthermore, we found a correlation of the heart rate with the gamma band in S1 in the absence of a reaction in wake and sleep for noxious stimulation. These findings confirm that somatosensory information, including nociception, is sensed and processed during sleep even in the absence of a behavioral response.

## Highlights

• Noxious and non-noxious stimuli are processed in the anterior cingulate and somatosensory cortices in natural sleep in mice.
• Noxious stimuli significantly increased heart rates in both wakefulness and NREM sleep when compared to non-noxious stimuli.
• Somatosensory stimulation in sleep evoked complex brain activity patterns in delta, alpha, and gamma frequency bands.
• Persistent evoked spectral shifts without awakening suggests acute somatic stimuli impair sleep restorative effects.
• Heart rate and gamma activity correlations differentiate between somatic stimuli, suggesting a novel pain assessment method.

## Introduction

Sleep is no longer described as a state of total disconnection from the environment (Hennevin et al., 2007). Auditory and visual stimuli have been shown to activate primary and supplementary cortical areas during sleep in humans (Andrillon et al., 2016, Sharon and Nir, 2018) and animals (Nir et al., 2015, Sela et al., 2020). Yet, evidence on somatosensory processing while asleep is sparse, particularly for noxious stimulation. Pain-induced sleep disruptions (Finan et al., 2013) suggest that pain can be processed during sleep. In chronic neuropathic pain, local cortical microarousals in non-rapid eye movement sleep (NREMS) occur more frequently resulting in a concomitant autonomic response and a higher vulnerability to stimulus-evoked awakenings (Cardis et al., 2021). In this respect, activation of the autonomic nervous system, reflected by heart rate changes, has been used clinically to assess pain perception in the awake state (Forte et al., 2022a, Forte et al., 2022b, Hilgard et al., 1974, Loggia et al., 2011, Möltner et al., 1990, Terkelsen et al., 2005, Tousignant-Laflamme et al., 2005). The same effect has also been observed in sleep (Chouchou et al., 2011). Furthermore, trained human participants confirm acute nociception during sleep (Mazza et al., 2012) further supporting the hypothesis that pain is processed during sleep.

However, studies investigating cortical pain processing are commonly performed in awake human subjects (Su et al., 2020) or in awake (Acuña et al., 2023) and deeply anesthetized animals (Kasanetz & Nevian, 2021). Despite activation of similar brain areas by noxious stimulation in the two above mentioned consciousness states, anesthesia is not equivalent to natural sleep. Pain studies in naturally sleeping subjects have shown preserved, yet distinct, activity patterns in pain-related brain areas. In humans, laser-evoked potentials display lower amplitudes in the cingulate cortex during NREMS compared to wake (Bastuji et al., 2012). The same trend was observed for cutaneous noxious electrical stimulation in the secondary somatosensory cortex (S2) (Kakigi et al., 2003), the primary somatosensory cortex (S1), motor cortex, cingulate cortex, insula and medial temporal areas (Wang et al., 2004). However, the opposite effect, i.e. higher amplitudes, was observed after median nerve stimulation in S1 and S2 (Kitamura et al., 1996) and in EEG recordings from rats (Shaw et al., 2006). Regardless of the discrepancies in the amplitudes of the somatic evoked potentials, these studies show that somatic stimulation during sleep recruits cortical areas belonging to the “pain matrix” (De Ridder et al., 2022, Legrain et al., 2011).

S1 and the anterior cingulate cortex (ACC) are crucial for the experience of pain as they are involved in the processing of its sensory and affective-motivational components, respectively (Kasanetz et al., 2021). Given that the pain pathways terminating in S1 and ACC can be anatomically and functionally separated, the sensory and the emotional experience of pain can be dissociated as demonstrated in cases of anterior cingulotomy (Agarwal et al., 2016).

We hypothesized that noxious stimulation during sleep can be processed in S1 and ACC. To test this hypothesis, we recorded heart rate and neural activity during the sleep-wake cycle in mice chronically implanted with electrodes in S1 and ACC. In NREMS, sensory stimulation elevated the heart rate, with noxious stimuli inducing larger increases and more awakenings than non-noxious stimuli. Somatosensory evoked potentials (SSEPs) derived from LFP recordings in S1 and ACC indicated that both cortices were activated in a sequential manner that was conserved during sleep. Stimulus-induced changes in neural oscillatory activity in the broad gamma (>30H) and alpha (11–16 Hz) ranges corroborated somatosensory information processing (Başar et al., 1999, Başar-Eroglu et al., 1996, Heid et al., 2020, Karakaş et al., 2001) during NREMS with different characteristics as compared to the awake state, while being similar for noxious and non-noxious stimuli. However, the correlation between gamma activity in S1, with the heart rate changes, distinguished noxious from non-noxious stimuli in wake and NREMS, in the absence of a behavioral response.

## Materials and methods

### Animals

Male C57BL/6J mice aged 6 weeks were grouped together, caged with food and water *ad libitum*, in a 12:12 light–dark cycle with lights on at 06:30 (corresponding to Zeitgeber Time 0 (ZT0)). At 12–14 weeks of age, 18 animals were implanted with tetrodes in the ACC and the S1HL, together with a frontal and a parietal electroencephalogram (EEG), a neck electromyogram (EMG) as well as an electrocardiogram (ECG) in the lower back. All recording electrodes were referenced to the ground screws, placed above the cerebellum. One animal had to be excluded from the study given it presented an anatomical malformation of the brain. Misplaced tetrodes were excluded post-hoc from the analysis. Following implantation, animals were kept housed together. Recordings started 14–16 days after the implantation surgery. All experiments were conducted after the approval of the cantonal veterinary office of Bern, Switzerland.

### Surgery for chronic neural recordings

Anesthesia was initially induced with 5 % isoflurane at a flow of 2 l/min of O2 and analgesia was achieved with an initial injection of 2.5 mg/kg, 0,5 mg/ml of carprofen. During surgery animals were maintained at 1.5 – 2 % isoflurane at a flow of 1.5 l/min of O2. Animals were fixed on a stereotaxic frame and 0.3 mm craniotomies were performed to implant tungsten tetrodes bilaterally in the ACC (AP: + 0.6, ML: +/- 0.3, DV: − 1.4 from brain surface) and the S1HL (AP: −0.1, ML: +/- 1.9; DV: −0.4). Craniotomies of 0.9 mm were used to insert stainless steel screws to measure the frontal EEG (AP: +2.5, ML: − 1.4), the parietal EEG (AP: −2.6, ML: − 2.5) and the ground (GND; AP: −5.2, ML: +/- 1.5). Finally, two bare-ended EMG coated sliver wires were sutured to the epaxial neck muscles to record muscle tone and two additional bare-ended EMG wires were positioned at the dorsal section of the quadriceps muscle to record the electrocardiogram (ECG). The GND screws on the cerebellum were used as reference. Tetrodes, EEG and GND screws as well as the head stage were held in place using dental cement (Tetric EvoFlow Light-Cure).

### Data acquisition

All electrophysiological signals were acquired and amplified using RHD2000 amplifier boards and digitized at a rate of 20′000 Hz by the Intan RHD USB Interface board (Intan Technologies, Los Angeles, CA). During the habituation and the recording sessions, animals were tethered to the recording setup. Animals were habituated to the recording setup 1 – 3 days previous to the first recording session. Mice were placed in an 8x8x12 cm red plexiglass cubicle on a von Frey grid. The von Frey grid was placed inside a chamber insulated with acoustic foam panels, where the researcher had access to the plantar surface of the hind paws, where the mechanical stimulation was applied. During the recordings, animals had no access to water or food and did not have nests in the cubicles. Recording sessions were performed on the second half of the light cycle. Thus, the first stimulation was performed between ZT6 and ZT7. One recording session consisted of 100 stimuli of one type (either Noxious (Nox) or Non-Noxious (NN)). The inter-trial interval as well as the stimulated hind paw were random. Each animal received a total of 200–300 stimuli per stimulation type in the span of 2 to 3 recording sessions. The experimenter marked stimuli that induced paw withdrawal, flinching or licking with a TTL pulse to the Intan RHD USB Interface board. To synchronize the stimuli with the electrophysiological data, either a 20 G needle (for the painful stimulation) or a yellow pipette tip (for non-painful stimulation) were attached to a load cell, which in turn was connected to the Intan RHD USB Interface board. This system allowed for the recording of voltage deflections evoked by changes in pressure on the loading cell. The recorded voltage from the loading cell was used to define the precise start and end of each stimulation.

### Histological verification of intracranial recording sites

After the recording experiments, animals were deeply anesthetized with 5 % isoflurane at a flow of 2 l/min of O2 followed by an intraperitoneal injection of 80/10 mg/kg Ketamine/Xylosine mixture. To confirm the tetrodes location, electrolytic lesions were performed by applying 2 s long 30 µA current 5 times per electrode. After transcardial perfusion of 4 % paraformaldehyde (PFA), heads were kept in PFA at 4 °C for 4 days. Brains were retrieved on day 5 and further post-fixed in PFA for 12 h at 4 °C. Brains were washed in PBS and sliced at 70 µm. Brain sections were stored in PBS. For imaging, brain sections were mounted with Mowiol ® 4–88 mounting medium and imaged to confirm the location of the tetrodes.

### Analysis

#### Stimulation detection

The voltage deflection signals of the stimuli were down-sampled at 1000 Hz. Using a custom-made software, the signal was displayed and the researcher marked the onset and offset of each stimulation.

#### Sleep scoring

The signals used for sleep scoring were the two EMG of the neck, the frontal EEG and the parietal EEG. We additionally displayed the ECG as an additional source of information (Suppl. Fig. 1A). The scoring of the different arousal states was done manually by the experimenter using a custom-made software written in Python. This software was designed to score without a scoring window, allowing for high temporal precision and precise marking transition periods and short-lived events (Suppl. Fig. 1B). Artifacts and movement-related noise were marked during the scoring and discarded a posteriori.

We defined “wake” as high muscle activity in the EMG (i.e. indicating active muscle engagement or contraction) and fast low-amplitude oscillations in both EEG channels (Suppl. Fig. 1A). The minimum accepted duration of a wake bout was 1 s, corresponding to a microarousal during NREMS (Suppl. Fig. 1B). NREMS was defined as slow-frequency high-amplitude fluctuations in the EEG and the absence of muscle activity. Rapid Eye Movement Sleep (REM) was defined by the absence of muscle tone (i.e. decrease or loss in normal muscle tension) and strong theta oscillations (4–8 Hz) in the EEG. Periods of the signal with mixed NREMS and wake characteristics occurring during wake were discarded. Movement-related noise was observed only during wake and consisted of synchronized slow oscillations (<1 Hz) in the EEG and EMG, while artifacts consisted of glitches and high frequency activity simultaneously occurring in the EEG and EMG.

#### Data preparation

EEG and local field potentials (LFP) were low-pass filtered at 300 Hz. EMG was bandpass filtered between 100 Hz and 500 Hz. LFP, EEG, EMG and ECG were all down-sampled to 1000 Hz. Filters for EEG, LFP and EMG were applied before down-sampling. ECG filters were applied after down-sampling. To process the ECG, slow oscillations were first removed, then, high frequency muscle activity smoothed with a Wiener filter using the Python command *scipy.signal.wiener*.

Stimuli were collected in data epochs that spanned from −10 s before the stimulation onset to + 20 s after the stimulation onset. In order to automatically detect outliers, a z-score on the mean of the time series (*stimulus*) for each stimulation (*z-score(stimulus)*) was calculated with the following formulae:

$$z-score\left(stimulus\right)=\frac{\mathrm{stimulus}-stimulusaverage}{\mathrm{stimulusstd}}$$

$$\mathrm{stimulus}=\frac{\left(\sum_{x=0}^{x=t}dataepoch\left(x\right)\right)}{t}$$

$$\mathrm{stimulusaverage}=\frac{\left(\sum_{n=1}^{n=N}stimulus\left(n\right)\right)}{N}$$

$$\mathrm{stimulusstd}=\sqrt{\frac{\sum_{n=1}^{n=N}\left({\mathrm{stimulus}}_{n}-mean\left(allstimuli\right)\right)}{N}}$$

where *t* is the total number of points constituting each data epoch, and *N* the total number of stimulations for that animal.

Any stimulation which z-score surpassed ± 3 std was eliminated.

#### Quantification of somatosensory evoked potentials and peaks detection

Somatosensory evoked potentials (SSEPs) were lowpass filtered below 20 Hz using the Python command *scipy.signal.filtfilt*. Baseline normalization was done by subtracting the mean of the pre-stimulus window (−0.3 to −0.1 s). Then, the resulting time-series were cut between −0.2 and 1 s.

We were interested in extracting peak amplitude and timing from the first and last voltage deflections of the SSEPs. The variance in the signal – considered as high frequency oscillations riding the slow voltage deflections of SSEPs – was high, impeding the accurate computation of the peak times. Thus, given the bell-like shape of the SSEPs voltage deflections, we applied a Gaussian fit to each voltage deflection to minimize the effect of the variance, consequently improving the detection of peak times. To do this, we first averaged the SSEPs for each recording site, independently of condition and animal, obtaining a general SSEP that we called “global SSEP”. Then, by visual inspection of global SSEPs, we set the boundaries for each peak and brain region (Suppl. Fig. 2A). Given that the global mean of SSEPs can be interpreted as the average SSEP, the boundaries were chosen based on the beginning and end of each oscillation (Suppl. Fig. 2A). Boundaries were the following: ACC 1st peak: [0, 200] msec; ACC last peak: [100, 700] msec; S1HL 1st peak: [0, 60] msec; S1HL last peak: [80, 500] msec. For each peak, condition and animal we applied a Gaussian fit using the Python command *scipy.optimize.curve_fit*. obtaining the peak times (Suppl. Fig. 2). Given that the amplitude of the fitted Gaussian was not the real recorded signal, we decided to average the values of the recorded signal around the peak time for a more accurate readout. To do so, we set a window around the peak time which duration was 10 % of the manually chosen boundaries of each fluctuation. However, if the width of the fitted Gaussian was larger than the manually chosen boundaries, the window to calculate the peak amplitude was 30 % instead of 10 %. Visual inspection of the data histograms confirmed that the variables were roughly normally distributed (not shown here).

#### Spectro-temporal data collection

Extraction of spectral features was performed using a custom-written Complex Wavelet Transform (CWT) function (Cohen, 2014). Power of 64 logarithmically spaced frequencies between 0.5 and 160 Hz were extracted for each trial using Complex Morlet Wavelets.

Slow wave activity was collected as the summed power of frequencies below 4 Hz over time.

For the spectro-temporal analysis, the spectrogram of each individual trial was computed. Then, the spectrograms were baseline normalized in decibels (dB) and later cut at the time points of interest. Stimulus evoked changes within the first 400 msec after the stimulation onset were considered phasic responses. While those lasting between 0.5 and 16 s were considered sustained. For the analysis of sustained responses, the spectrograms of stimuli without a behavioral response were subtracted from the spectrograms with a behavioral response per animal and condition. If an animal presented one single trial for a given condition, the data from that animal was excluded from the final analyses of that condition.

#### Heart rate analysis

From the 17 animals, 14 presented clear ECG signal in wake and NREMS. Thus, we used these 14 animals for any analysis involving heart rate measures. R peaks of the ECG were detected using the Python command *scipy.signal.find_peaks* and the peak times were used to create a binary signal to compute the heart rate (HR). From the binary signal, a second signal with the R-R distance for each beat time was created to compute the heart rate variability (HRV). Convolution of the binary signal and the R-R distance signal with a unit kernel of 4 s was used to retrieve the HR and the HRV, respectively. Baseline normalization of HR was performed using % change calculated as

$$\%change=\frac{{\mathrm{HR}}_{t(-2,16)}-{\mathrm{HR}}_{t(-2,0)}}{{\mathrm{HR}}_{t(-2,0)}}\times 100$$

Baseline normalization of HRV was performed by z-scoring:

$$z-score=\frac{{\mathrm{HRV}}_{t(-2,16)}-{\mathrm{mean}(HRV}_{t\left(-2,0\right)})}{\mathrm{standarddeviation}({\mathrm{HRV}}_{t(-2,0)})}$$

In both equations *HR(baseline)* and *HRV(baseline)* represent the baseline window of each respective signal.

A window between 2 and 10 s, defined by the begin and end of HRV increases, was used to calculate the mean HR and HRV.

#### Correlations of heart rate with alpha and broad gamma frequency bands

The heart rate-power correlations were performed with the unprocessed frequency power of neural signals and the unprocessed heart rate signal for each animal. Trial-by-trial correlations of HR with either alpha (8 – 15 Hz) or broad gamma (30 – 160 Hz) frequency bands were done by feeding the mean value of a window (alpha: 0–3 s for the early window and 6–16 s for the late window; gamma: 0–2 s for the early window and 6–16 s for the late window) to the Python function to compute Pearson correlations *scipy.stats.stats.pearsonr*. The resulting Pearson correlation coefficients exceeding ± 0.98 were defined as outliers and excluded from the analysis. Data from animals with fewer than five stimuli in a condition were excluded from the final analyses of that condition.

### Statistics

All data are presented as mean ± SEM as the average of all animals unless otherwise specified. Stimuli were pooled according to condition, independently on which recording session the condition was observed. When the average of any signal needed to be computed, we first computed per condition within each animal, and then averaged across animals for the final plots. ANOVA models were created with the Python method to evaluate a linear regression model *statsmodels.formula.api.ols* and were later fed to the Analysis of Variance of linear models *statsmodels.api.sm.stats.anova_lm*. All post-hoc analysis as well as comparisons between two groups used the Python library algorithm to compute t-tests of two independent samples *scipy.stats.ttest_ind*.

#### Time series and time–frequency statistics

Statistics on the time domain were performed by computing the difference of two time series (each of which belonging to a different condition) and feeding the result to the Python algorithm *scipy.stats.ttest_1samp*. The resulting statistics were corrected for multiple comparison using false discovery rate (FDR) correction with the Python function *statsmodels.stats.multitest.fdrcorrection*. This analysis is also used to confirm the presence of evoked potentials in Supplementary Fig. 7.

For time–frequency statistics, all spectrograms were first smoothed, with a normalized 2-D Gaussian, using the Python algorithm for convolution of two signals *scipy.signal.convolve2d*. Time series statistics, as described on the above paragraph, were applied for each frequency vector.

#### SSEP peaks

We analyzed the peak time and amplitude within each brain area using a two-way ANOVA where the independent factors were “stimulation type” and “arousal state”.

#### Heart rate statistics

To first assess whether the stimulation type or the arousal state had any influence on stimulation-induced changes in HR and HRV, we performed a two-way ANOVA using the two independent factors “stimulation type” and “arousal state” (Table 1). Given that both independent factors had an effect on HR and HRV changes, we decided to evaluate the effect of “behavioral response” and “arousal state” on HR and HRV in each type of stimulation separately. Thus, we used a two-way ANOVA with “behavioral response” and “arousal state” as independent factors.

**Table 1:** Results of a 2-way ANOVA to assess the effects of Stimulation type and Arousal and their interactions on HR. Statistics (F) and p-values (p) for HR corresponding to Fig. 2A. Calculated from the mean of an 8-second window (see methods). *: p < 0.05, **: p < 0.01.

	**HR**	
	**F**	**p**
Stim. Type	9.65	0.003 **
Arousal	44.59	0.1x10^-9^ **
Stim. Type: Arousal	5.70	0.02 *

To evaluate the within-group statistics for the correlations of HR with frequency bands, we used the Python algorithm *scipy.stats.ttest_1samp* and later corrected for multiple comparison with the FDR algorithm *statsmodels.stats.multitest.fdrcorrection*.

## Results

### Mechanosensory stimuli evoke behavioral responses in sleep and wake

**Fig. 1:**
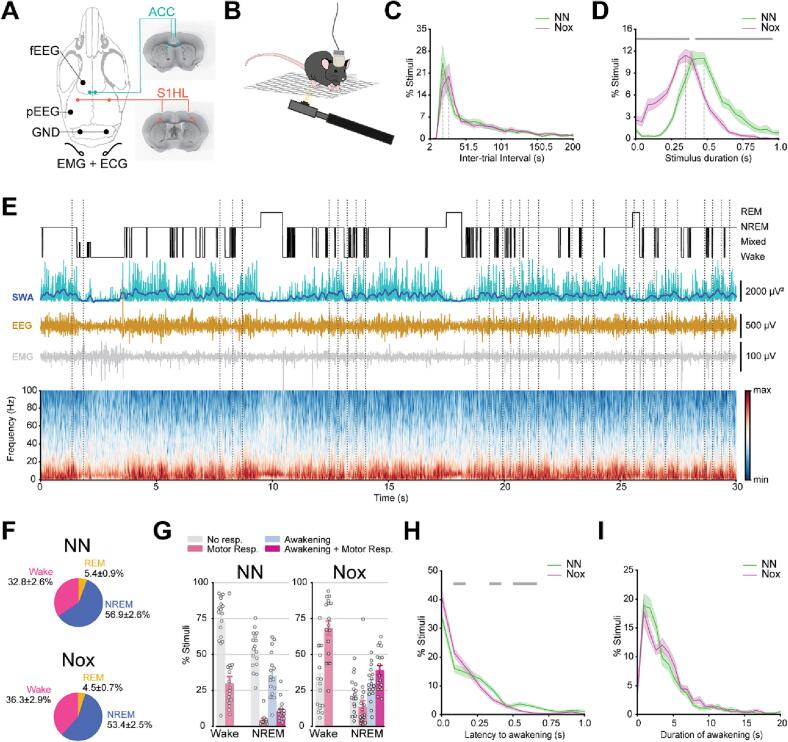
Setup and behavioral characterization. A, Recording sites (left) and examples of electrolytic lesions (right) in ACC (top) and S1HL (bottom). B, Illustration of the experimental setup with an animal on a grid receiving stimulation to the hind paw using a dynamic von Frey device. C, Distributions of the inter-trial intervals (ITIs). Dashed lines indicate the most common ITIs. D, Distributions of stimulation durations. Vertical dashed lines indicate the most common stimulus duration. Grey horizontal bar, p < 0.05, FDR corrected. E, Example recording of 30 min of a NN session for one animal. From top to bottom: Hypnogram, raw slow wave activity (SWA, cyan) and smoothed SWA (dark blue), EEG, EMG, spectrogram of the EEG. Vertical dotted lines indicate stimuli onset. F, Distributions of stimuli in the different arousal states for the recording period between ZT6 and ZT10. G, Behavioral responses. H, Distributions of time to awakening from stimulation onset for those stimuli in NREMS that evoked awakening. Grey horizontal bar, p < 0.05, FDR corrected. I, Distribution of time awake after stimuli onset for those stimuli in NREMS that evoked awakening. Data is represented as mean ± SEM. N = 17. (For interpretation of the references to colour in this figure legend, the reader is referred to the web version of this article.)

We aimed to investigate the processing of somatosensory information and, particularly, nociception, during sleep and wake. To accomplish this, we simultaneously recorded local field potentials (LFP) in the hindlimb area of S1 (S1HL) and the ACC together with EEG and ECG, in freely moving male mice placed on a grid, which received mechanical stimulation of the plantar sole (Fig. 1A, B). Stimuli were applied with a needle or a yellow pipette tip mounted on a dynamic von Frey device for the noxious (Nox) and the non-noxious (NN) stimulation, respectively. Sessions during which non-noxious stimuli were delivered lasted 4 h 24 min ± 15 min, and sessions of noxious stimulation, 4 h 58 min ± 11 min. Stimuli were delivered at random intervals between 17 s and 51 s. The inter-trial intervals were equally distributed for both stimulus types (Fig. 1C, NN: 35 ± 5 sec; Nox: 35 ± 3 sec; T(32) = 0.07, p = 0.939). Repeated manual mechanical stimulation varied in duration and was significantly shorter for noxious than for non-noxious stimuli due to shorter response latencies to noxious stimuli (Fig. 1D, NN: 460 ± 21 msec; Nox: 350 ± 25 msec; T(32) = 3.2, p = 0.002). Animals underwent the natural stages of wake and sleep (Fig. 1E). With our stimulation paradigm, the percentage of time spent in each arousal state was comparable to undisturbed recordings (Suppl. Fig. 3A). However, a thorough quantification of different sleep aspects revealed that the stimulations caused sleep fragmentation. In non-noxious and noxious sessions, there were relatively more bouts in wake, and less bouts of NREMS and REMS (Suppl. Fig. 3B) together with a larger fraction of longer wake bouts and shorter NREMS and REMS bouts (Suppl. Fig. 3C). The mean bout duration of wake and NREMS was greatly decreased in non-noxious and noxious sessions, while REMS mean bout duration was not affected (Suppl. Fig. 3D). These effects were reflected in a leftward shift of the distribution of bout durations (Suppl. Fig. 3E). Nevertheless, the number of stimuli delivered in each arousal state reflected the prevalence of the corresponding arousal state in the light phase (Fig. 1F and Suppl. Fig. 3). Given the low number of stimuli performed during REMS, we excluded this sleep state from further analysis.

Stimuli that did not evoke either awakening or motor responses were classified as “no response”. Accordingly, we classified a stimulation as causing a “behavioral response” if it resulted in a change of arousal state (i.e. awakening as detected with the EEG and EMG) and evoked a nocifensive behavior as observed by the experimenter. Paw withdrawal, flinching or licking of the stimulated paw were categorized as a “motor response”. For stimuli delivered during NREMS, the subsequent presence of wake EEG and EMG characteristics within 5 s after stimulation onset were classified as awakening, which could coexist with a “motor response” (Fig. 1G and Suppl. Fig. 4). As expected, noxious stimuli in wake evoked motor responses more often than non-noxious stimuli (Nox: 68.4 ± 4.9 %, NN: 29.9 ± 4.7 %, T(32) = 5.4, p = 4.6x10^-6^). In NREMS, the occurrence of motor responses were not different between stimuli (Nox: 13.7 ± 4.1 %, NN: 4.3 ± 1.2 %, T(27) = 1.7, p = 0.08), but noxious stimulation caused simultaneous motor response and awakening more often as compared to non-noxious stimuli (Nox: 39.1 ± 3.3 %, NN: 10.2 ± 1.7 %, T(32) = 7.4, p = 1.7x10^-8^). Overall, the lack of “behavioral reactions” during NREMS were detected more often upon non-noxious compared to noxious stimulation (Nox: 21.9 ± 2.9 %, NN: 52.9 ± 3.1 %, T(32) = -7.1, p = 4.4 x10^-8^). However, the rate of stimulus-evoked awakenings remained equal for both stimuli (Nox: 29.5 ± 2.8 %, NN_._: 35.1 ± 3.7 %, T(32) = -1.1, p = 0.251). Yet, animals awoke faster after noxious compared to non-noxious stimulation (Fig. 1H) but the duration of the stimulus-evoked awake bouts did not differ between stimulus types (Fig. 1I). We conclude that noxious stimuli had a stronger effect on animal behavior and lead in total to more frequent awakenings.

For the subsequent analysis, we grouped behavioral responses as “No Response” or “Response”. In wake, “Response” trials corresponded to stimuli that evoked a motor response. In NREMS, stimuli that caused an awakening, with or without an accompanying motor response, were classified as “Response”. “No Response” trials in NREMS consisted of stimuli that did not evoke any type of behavioral response.

### Autonomic responses confirm somatosensory processing during NREMS

**Fig. 2:**
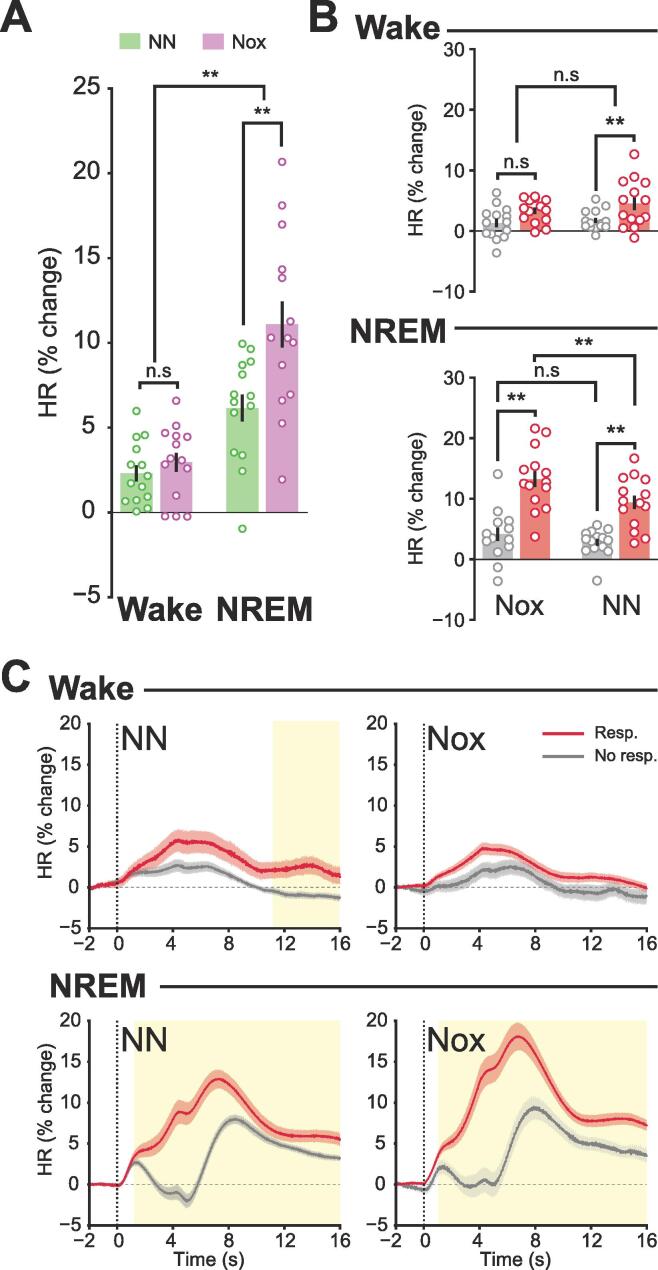
Stimulus-evoked changes in heart rate. A, Heart rate (HR) in percent change from baseline. B, Bar graphs of the mean HR of an 8 s window between 2 and 10 s after stimulation onset. C, Time course of the percent change in HR. Yellow boxes represent p < 0.05, FDR corrected. Each data point represents one animal (N = 14). Data were represented as mean ± SEM. *, p < 0.05; **, p < 0.001; n.s., not significant. See Table 1, Table 2 for ANOVA statistics. (For interpretation of the references to colour in this figure legend, the reader is referred to the web version of this article.)

To assess how sensory stimuli activated the autonomic nervous system, as a first indication of stimulus perception (Forte et al., 2022a, Forte et al., 2022b, Hilgard et al., 1974, Loggia et al., 2011, Möltner et al., 1990, Terkelsen et al., 2005, Tousignant-Laflamme et al., 2005), we evaluated stimulus-evoked changes in heart rate. The baseline heart rate of animals was 565 ± 12 beats per minute (bpm) during wake and 480 ± 14 bpm during NREMS. A 2-way ANOVA was used to assess the effect of “arousal state” and “stimulation type” on stimulus-evoked heart rate changes (Table 1), which confirmed that increases in heart rate were associated with both factors. This implied that both stimulus types induced heart rate increases in both arousal states (Fig. 2A). Furthermore, the analysis revealed a significant interaction effect, where noxious stimulation led to larger increases in heart rate compared to non-noxious stimulation in NREMS (Nox: 11.07 ± 1.3 %, NN: 6.1 ± 0.8 %, T(26) = -3.03, p = 0.005) but not in wake (Nox: 2.9 ± 0.5 %, NN: 2.3 ± 0.4 %, T(26) = -0.8, p = 0.4) (Table 1). Stimulus-induced heart rate increases were significantly larger in NREMS compared to wake for both noxious (wake: 2.9 ± 0.5 %, NREMS: 11.07 ± 1.3 %, T(26) = 5.3, p = 0.1x10^-6^) and non-noxious stimulation (wake: 2.3 ± 0.4 %, NREMS: 6.1 ± 0.8 %, T(26) = 4, p = 0.0004) (Fig. 2A).

After confirming that both noxious and non-noxious stimuli activate the autonomic nervous system in wake and NREMS, we analyzed the effect of “behavioral response” and “arousal state” for each stimulation type separately. A 2-way ANOVA revealed a strong effect of both factors on heart rate increases evoked by non-noxious and noxious stimulation (Table 2).

**Table 2:** Results of a 2-way ANOVA on HR to assess the factors Arousal and Behavioral Response following non-noxious (NN) and noxious (Nox) stimulation. Statistics (F) and p-values (p) for HR of an 8 s window corresponding to Fig. 2C. *: p < 0.05, **: p < 0.01.

	**NN**		**Nox**	
	**F**	**p**	**F**	**p**
Arousal	11.48	0.001 **	31.08	8.94x10^-7^ **
Behav. Resp	28.77	2x10^-6^ **	23.15	1.32x10^-5^ **
Arousal: Behav. Resp	5.21	0.026 *	8.91	0.004 **

Compared to non-noxious stimuli that did not evoke a behavioral response, those that did resulted in greater increases in heart rate in both wake (Resp: 4.5 ± 1, NoResp: 1.8 ± 0.4, T(26) = -2.2, p = 0.03) and NREMS (Resp: 9.4 ± 1, NoResp: 2.8 ± 0.5, T(26) = -5.1, p = 0.2x10^-6^) (Fig. 2B). Yet, given the dependency between both independent factors, stimulus-induced heart rate rises were more pronounced in NREMS compared to wake in the presence of overt behavioral responses (NREMS: 9.4 ± 1, wake: 4.4 ± 1, T(26) = 3.1, p = 0.003) but not in their absence (NREMS: 2.8 ± 0.5, wake: 1.8 ± 0.4, T(26) = 1.2, p = 0.2) (Fig. 2B).

For noxious stimulation the interdependence of the two independent factors was more complex. Behavioral-response-evoking stimuli increased the heart rate in NREMS (Resp: 13.2 ± 1.3, NoResp: 4.1 ± 1, T(26) = -5.1, p = 0.2 x10^-6^) but not in wake (Resp: 3.3 ± 0.5, NoResp: 1.4 ± 0.7, T(26) = -2.04, p = 0.0506) (Fig. 2B). Furthermore, we observed an interaction between the stimulation type and the arousal state, which manifested as noxious stimuli evoking overt behavioral responses that increased the heart rate significantly more in NREMS than in wake (NREMS: 13.2 ± 1.3, wake: 3.3 ± 0.5, T(26) = 6.8, p = 0.7x10^-8^). This effect was not present in the absence of overt behavioral responses (NREMS: 4.1 ± 1, wake: 1.4 ± 0.7, T(26) = 1.9, p = 0.056) (Fig. 2B).

Since larger heart rate increases were associated with the presence of a behavioral response, and these responses (i.e. awakening from NREM or motor responses in wake) spanned a few seconds, we analyzed the time series of the stimulus-induced heart rate changes (Fig. 2C). Heart rate responses to both noxious and non-noxious stimulation were similar between both behavioral responses. However notable differences appeared between wakefulness and NREMS. During wakefulness, stimulus-induced heart rate increases were brief, whereas in NREMS, they persisted for a longer duration, even when there were no overt behavioral responses. The sustained increase in heart rate during NREMS did not reach baseline wakefulness levels for neither stimulation type (NN_NoResp_: 490 ± 16 bpm, NN_Resp_: 511 ± 16 bpm, Nox_NoResp_: 490 ± 13 bpm, Nox_Resp_: 521 ± 13 bpm). These results show that stimuli delivered during NREMS cause changes in the autonomous reaction of the animal that are not observable through behavior alone, emphasizing the usefulness of multiple measures to infer pain perception.

Heart rate variability has also been proposed to be altered by noxious events (Forte et al., 2022a, Forte et al., 2022b), although reports have been inconclusive (Koenig et al., 2014). Thus, we evaluated the changes in heart rate variability in the context of acute noxious and non-noxious stimulation (Suppl. Fig. 5). The baseline of heart rate variability was 11.09 ± 1.03 msec in wake and 12.48 ± 0.97 msec in NREMS. Using a 2-way ANOVA we revealed that changes in the heart rate variability were exclusively associated with the arousal state and had no interactive effect with the stimulation type (Suppl. Table 1). Stimulus-induced increases in heart rate variability were more prominent in NREMS compared to wake for both noxious (wake: 1.2 ± 0.3 %, NREMS: 4.5 ± 1.2 %, T(26) = 2.4, p = 0.02) and non-noxious stimulation (wake: 0.4 ± 0.3 %, NREMS: 4.5 ± 1.2 %, T(26) = 3.8, p = 0.0006) (Fig. 2B). Following the same analysis steps as for the analysis of heart rate, we used a 2-way ANOVA for each stimulation type with the independent factors “behavioral response” and “arousal state” (Suppl. Table 2). The increase in heart rate variability was associated with the arousal state, with larger rises in NREMS compared to wake for both non-noxious (NREMS: 7.6 ± 1.4, wake: 0.5 ± 0.3, T(26) = 4.7, p = 0.7x10^-5^) and noxious stimulation (NREMS: 6.7 ± 1.6, wake: 0.9 ± 0.4, T(26) = 3.2, p = 0.003) (Suppl. Fig. 5A). However, in the presence of a behavioral response, a relationship with the arousal state was observed for non-noxious (NREMS: 3.6 ± 1, wake: 0.07 ± 0.5, T(26) = 2.8, p = 0.007) but not for noxious stimulation (NREMS: 3.6 ± 1.1, wake: 1.3 ± 0.3, T(26) = 1.8, p = 0.07). An association between behavioral response and heart rate variability was observed only for non-noxious stimuli (Suppl. Table 2). In NREMS, non-noxious stimuli that evoked a behavioral response increased heart rate variability more than those that did not (React: 3.6 ± 1, NoReact: 7.6 ± 1.4, T(26) = 2.2, p = 0.03). This pattern was not seen in wakefulness (React: 0.07 ± 0.5, NoReact: 0.5 ± 0.3, T(26) = 0.6, p = 0.4) (Fig. 2D,F). The time evolution of the heart rate variability was similar for both stimulation types, with striking differences between wakefulness and NREMS. In the latter, stimuli without a behavioral response increased the heart rate variability significantly more than those with a behavioral response (Suppl. Fig. 5C).

Given that heart rate primarily reflects sympathetic activation, and heart rate variability, parasympathetic activation, and considering that these two measures significantly affected each other (Kazmi et al., 2016, Sacha, 2014), we evaluated their mutual influence following stimulation (Suppl. Fig. 6). In NREMS, larger increases in heart rate variability tended to co-occur with reduced heart rate responses for stimuli that did not cause awakening. However, when animals awoke, the changes in heart rate variability were less pronounced, and increases in heart rate were greater (Suppl. Fig. 6A). These findings imply that heightened parasympathetic activity evoked by a stimulus may suppress sympathetic activation, preventing awakening from NREMS (Goldberger et al., 2001). This effect was not observed in the wake state (Suppl. Fig. 6B). Therefore, it appears that parasympathetic activation in response to stimulation during sleep may serve as a mechanism to preserve sleep continuity.

### Noxious and non-noxious somatosensory stimuli reach ACC and S1 during NREMS and can be distinguished

After confirming the activation of the autonomic nervous system by noxious stimulation, we evaluated whether somatic stimuli also activate cortical areas. Since LFPs are the linear sum of the neural inputs (local as well as long-range axonal terminals) around the location of the intracranial recording electrode and the EEG is a linear weighted combination of the activity of multiple, more spatially diverse, brain areas (Buzsáki et al., 2012, Cohen, 2017, Herreras, 2016), we used the LFP to infer the strength of cortical recruitment and the EEG to investigate the confluent activity of fronto-parietal regions.

**Fig. 3:**
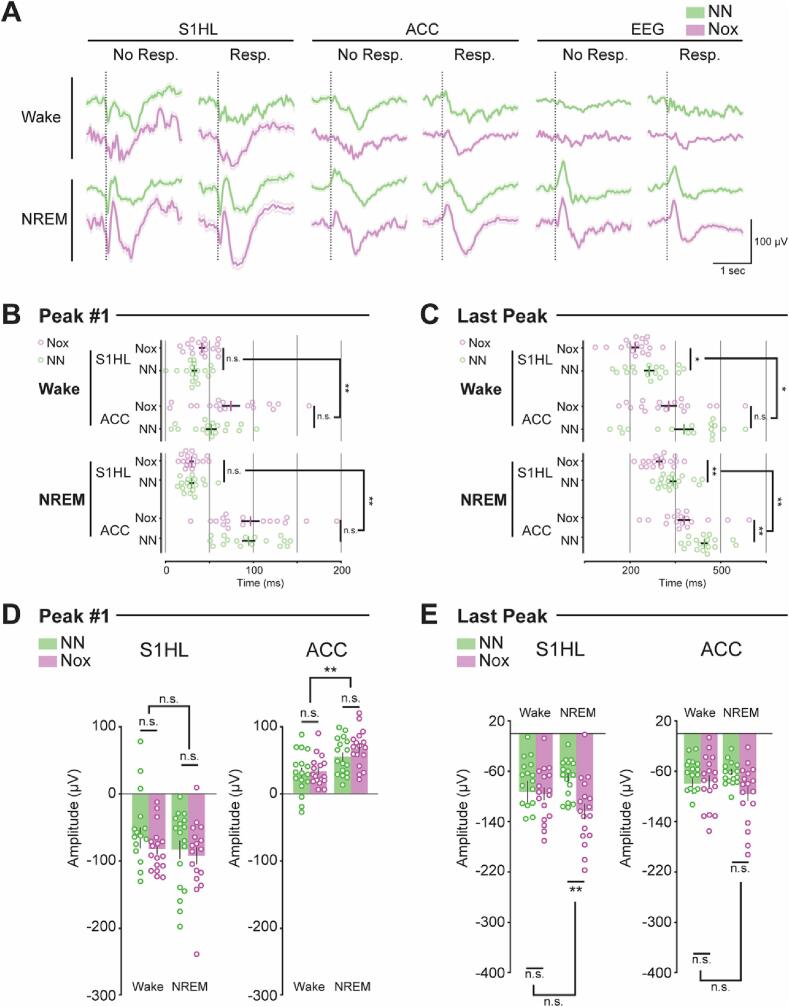
Analysis of somatosensory evoked potentials. A, SSEPs for each recording site, divided by type of stimulation, arousal state and behavioral response. B, Latencies to the first peak. C, Latencies to the last peak. D, Amplitude of first peaks. E, Amplitude of last peaks. Data were represented as mean ± SEM. N = 17. *, p < 0.05; **, p < 0.01. See Table 3 for statistics.

Somatic stimuli evoked somatosensory evoked potentials (SSEPs) in S1HL and ACC in both wake and NREMS for all response conditions (Fig. 3A and Suppl. Fig. 7). These results confirmed that noxious and non-noxious stimuli reached cortical areas across arousal states even in the absence of an overt behavioral response. Large biphasic SSEPs in the EEG were observed in NREMS (Fig. 3A), which were largely suppressed in the wake state (Suppl. Fig. 7). Thus, external stimuli generated strong synchronized temporal dynamics in multiple fronto-parietal regions during NREMS, but not during wakefulness.

The global averaged SSEPs exhibited a rapid first voltage deflection (positive in ACC and negative in S1HL), and a second negative deflection (Suppl. Fig. 2A). It is thought that the first peak can be attributed to the thalamo-cortical relay of sensory information, and later peaks result from the recruitment of local feedback circuits (Thorpe et al., 2021, Woodman, 2010). EEG studies in humans during wake show that the latencies to these peaks depend on the stimulation type (Thorpe et al., 2021, Woodman, 2010). Thus, this measurement has the potential to carry information about the nature of the stimulation. To test this hypothesis in our recordings we manually defined the peak boundaries of the global averaged SSEPs to fit a Gaussian and derive the peak time, as well as the peak amplitude (Suppl. Fig. 2). Given the anatomical and functional differences of ACC and S1HL, and the different neural mechanisms involved in the generation of the first and last SSEPs peaks (Thorpe et al., 2021, Woodman, 2010), we investigated whether the type of stimulation and the arousal state would differentially modulate the peak amplitudes and times of the first and last peaks in each brain area. To this end, a 2-way ANOVA with the independent factors “Stimulation type” and “Arousal state” was applied to each peak of each brain region (Table 3). With these results, we first assessed the temporal order in the recruitment of S1 and ACC indicating the flow of somatosensory information (Table 3). The latency to the first peak in wake revealed that feed-forward thalamocortical circuits recruited first S1HL and then ACC for both noxious (S1HL: 41.2 ± 3.3 msec, ACC: 74.4 ± 10.3 msec, T(32) = 2.9, p = 0.005) and non-noxious stimuli (S1HL: 32.7 ± 3.1 msec, ACC: 51.9 ± 6.2 msec, T(32) = 2.6, p = 0.01) (Fig. 3B). These temporal dynamics were preserved in NREMS for both noxious (S1HL: 29.3 ± 2.3 msec, ACC: 96.6 ± 10.3 msec, T(32) = 6.1, p = 0.7x10^-8^) and non-noxious stimuli (S1HL: 29.5 ± 2.7 msec, ACC: 94.7 ± 7.6 msec, T(32) = 7.7, p = 0.6x10^-10^) (Fig. 3B). The latency to the last peak was used to assess the recruitment of local feedback circuits (Fig. 3C). We found that local circuits in S1HL were recruited before local circuits in ACC in both wake (S1HL_Nox_: 217.4 ± 12.7 msec, ACC_Nox_: 327.4 ± 26.6 msec, T(32) = 3.6, p = 0.001; S1HL_NN_: 263.4 ± 16.9 msec, ACC_NN_: 377.6 ± 32.4 msec, T(32) = 3.03, p = 0.004) and NREMS (S1HL_Nox_: 296.3 ± 10.8 msec, ACC_Nox_: 378.1 ± 20.3 msec, T(32) = 3.4, p = 0.001; S1HL_NN_: 342.3 ± 11.1 msec, ACC_NN_: 444.8 ± 11.8 msec, T(32) = 6.1, p = 0.7x10^-8^). These results demonstrated that the computation flow, measured as the order of cortical recruitment, was preserved across arousal states and followed a temporal structure by first recruiting S1HL and then ACC.

**Table 3:** Results of 2-way ANOVA on the amplitude and latency of each peak for the factors Stimulation type and Arousal.

		**AMPLITUDE**			
		**Peak #1**		**Last peak**	
		**F**	**p**	**F**	**p**
**ACC**	Stim. Type	1.16	0.2	1.53	0.2
	Arousal	20.07	0.1x10^-4^ **	0.003	0.9
	Stim. Type: Arousal	0.61	0.4	2.268	0.1

**S1HL**	Stim. Type	1.47	0.2	5.19	0.02 *
	Arousal	1.75	0.1	0.03	0.8
	Stim. Type: Arousal	0.13	0.7	3.95	0.04 *

		**TIME**			
		**Peak #1**		**Last peak**	
		**F**	**p**	**F**	**p**
**ACC**	Stim. Type	2.01	0.1	7.84	0.005 **
	Arousal	14.21	0.0002 **	7.98	0.005 **
	Stim. Type: Arousal	1.42	0.2	2.26	0.1
**S1HL**	Stim. Type	1.67	0.1	10.82	0.001 **
	Arousal	5.45	0.021 *	32.21	0.8x10^-9^ **
	Stim. Type: Arousal	1.77	0.1	0.0003	0.9

Statistics (F) and p-values (p) for peak time and amplitude. *: p < 0.05, **: p < 0.01.

We then assessed whether noxious and non-noxious stimuli recruited S1HL and ACC differently. The type of stimulation had an effect on the last peak (Table 3). Noxious stimuli recruited local feed-back circuits in S1HL earlier than non-noxious in both wake (Nox: 217.4 ± 12.7 msec, NN: 263.4 ± 16.9 msec, T(32) = -2.1, p = 0.04) and NREMS (Nox: 296.7 ± 10.9 msec, NN: 342.3 ± 11.1 msec, T(32) = -2.8, p = 0.007) (Fig. 3C). Recruitment of local feed-back circuits in ACC by noxious stimuli was also faster than by non-noxious stimuli in NREMS (Nox: 378.1 ± 20.3 msec, NN: 444.8 ± 11.8 msec, T(32) = -2.7, p = 0.009). This timing difference was not observed in wake (Nox: 327.4 ± 26.6 msec, NN: 377.6 ± 32.4 msec, T(32) = -1.1, p = 0.2) (Fig. 3C).

Lastly, we evaluated the effect of the internal state of the animal (wake vs. NREMS) at the time of the stimulation on the latencies to the peaks (Table 3). Latencies to the first peak following noxious stimulation were not significantly different between arousal states in ACC (wake: 74.4 ± 10.3 msec, NREMS: 96.6 ± 10.3 msec, T(32) = 1.4, p = 0.1) (Fig. 3B). In S1HL, noxious stimulation during NREMS resulted in an advanced first peak compared to wake (wake: 41.2 ± 3.3 msec, NREMS: 29.3 ± 2.3 msec, T(32) = 2.8, p = 0.008) (Fig. 3B). Interestingly, the first peaks evoked by non-noxious stimulation were affected differently by the arousal state. ACC recruitment was faster in wake then in NREMS (wake: 51.9 ± 6.2 msec, NREMS: 84.4 ± 8.2 msec, T(32) = 4.2, p = 0.0001), while S1HL activation was equally fast across arousal states (wake: 32.7 ± 3.1 msec, NREMS: 29.5 ± 2.7 msec, T(32) = 0.7, p = 0.4). Latencies to the second peak were slower in NREMS compared to wake in S1HL for noxious (wake: 217.4 ± 12.7 msec, NREMS: 296.7 ± 10.8 msec, T(32) = -4.5, p = 0.6x10^-6^) and non-noxious stimuli (wake: 263.5 ± 16.9 msec, NREMS: 342.3 ± 11.1 msec, T(32) = -3.7, p = 0.0006) (Fig. 3C). The second peaks observed in ACC did not show any differences between wake and NREMS for either noxious (wake: 327.4 ± 26.6 msec, NREMS: 378.1 ± 20.3 msec, T(32) = 1.4, p = 0.1) or non-noxious stimuli (wake: 377.6 ± 32.4 msec, NREMS: 444.8 ± 11.8 msec, T (32) = 1.9, p = 0.06) (Fig. 3C). In summary, noxious stimuli recruited ACC with a similar time lag in wake and NREMS, but recruited S1HL more rapidly during NREMS. In contrast, non-noxious stimuli recruited S1HL with the same timing in both wake and NREMS but recruited ACC later in NREMS than in wake. The last peak appeared later in NREMS than in wake only in S1HL, while ACC showed no difference in timing. Thus, noxious stimulation causes an earlier recruitment of cortical pain-processing areas than non-noxious stimuli.

We additionally evaluated the recruitment strength of S1HL and ACC circuits, measured as the peak amplitude (Fig. 3D,E), which reflects the degree of neuronal synchronization of cortical areas (Table 3). Amplitude differences of the first peak were associated with the arousal state in ACC as larger amplitudes were evoked in NREMS as compared to wake by both noxious (wake: 27.3 ± 4.3 µV, NREMS: 57.2 ± 5.4 µV, T(32) = -4.1, p = 0.0002) and non-noxious stimuli (wake: 25.7 ± 6.2 µV, NREMS: 46.6 ± 5.3 µV, T(32) = 2.4, p = 0.01) (Fig. 3D). The stimulation type was associated with amplitude differences of the last peak in S1HL only and showed an interactive effect with the arousal state as non-noxious stimulation evoked significantly smaller amplitudes in NREMS (NN: −58.9 ± 6 µV, Nox: −103.2 ± 10.4 µV, T(32) = -3.5, p = 0.001), but not in wake (NN: −77.5 ± 14.9 µV, Nox: −80.5 ± 8.4 µV, T(32) = -0.1, p = 0.8) (Fig. 3E). Noxious stimulation did not evoke different amplitudes in any arousal state. In conclusion, the recruitment strength of S1HL and ACC was independent of the stimulation type. However, the lower amplitude of the second peak in S1HL observed only for non-noxious stimulation during NREMS, might reflect a cortex-dependent gating effect for low-saliency stimulations, such as non-noxious stimuli.

In conclusion, these results confirm that somatosensory information can reach (first peak) and activate (last peak) S1HL and ACC in NREMS, as in wake. Furthermore, the computational flow of somatosensory information first recruits S1 and then ACC, a temporal order preserved across arousal states. Additionally, the effects of the arousal state and the stimulation type on the peak latencies indicate that different stimulation types can be detected during NREMS.

### Stimulus-evoked spectro-temporal dynamics suggest sensory processing during NREMS

Having established that nociceptive stimuli reach cortical areas during NREMS, we next studied their impact on rhythmic neuronal network activity. By analyzing the EEG and the LFP of ACC and S1HL in the spectral domain, we gained insight into the frequency dynamics of the local circuits. This approach is informative because different frequency bands have been associated with different cognitive processes (Başar et al., 1999, 2001) and are preserved across species (Buzsáki et al., 2013).

**Fig. 4:**
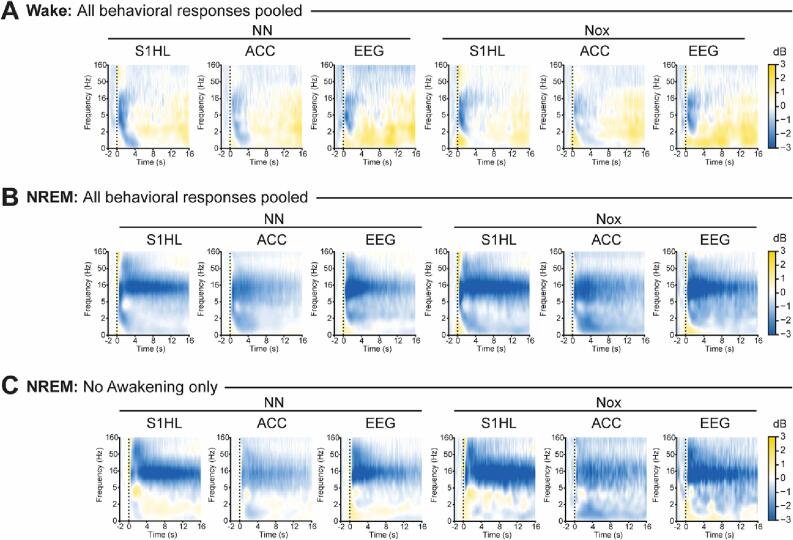
Descriptive spectral decomposition of stimulus-evoked oscillatory activity. A, Grand average spectrograms pooled independently of the behavioral response for wake. B, Grand average spectrograms pooled independently of the behavioral response for NREMS. C, Grand average spectrograms of stimuli in NREMS that did not evoke an awakening. A-C, Vertical dotted lines represent the stimulation onset. N = 17.

Stimulus-averaged spectrograms revealed significant differences between wake and NREMS, but very similar spectro-temporal profiles between non-noxious and noxious stimuli (Fig. 4A,B). In all conditions, stimuli increased gamma power (>30 Hz) immediately following stimulation onset, consistent with the established role of gamma in sensory processing (Başar-Eroglu et al., 1996, Karakaş et al., 2001). During wake, stimuli evoked a transient decrease of frequencies below 16 Hz (Fig. 4A), possibly indicating fast and directed attention to the location of the stimulation (8–16 Hz) (Ikkai et al., 2016, Magosso et al., 2019, Wöstmann et al., 2021). Following the initial desynchronization of low frequencies (<16 Hz), a generalized rebound extended up to 16 s for both stimulation types, potentially denoting sustained attention (8–16 Hz) (Fransen et al., 2016, Sobolewski et al., 2011) and stimulus processing (<4Hz) (Hauck et al., 2015).

During NREMS, all recording sites and stimulation types, showed a biphasic response in time in the gamma range characterized by an initial transient increase, time-locked to the stimulation onset that evolved into a depression for 3 to 4 s and a subsequent, diffuse second increase starting between 4 and 6 s after stimulation onset (Fig. 4B). The first activation is thought to primarily reflect sensory processes driven by bottom-up mechanisms. The second, diffuse activation, mediated by top-down mechanisms, reflects either perceptual or cognitive processes (Karakaş et al., 2001). Stimuli in NREMS additionally evoked a desynchronization of low frequencies (<16 Hz), which was bimodal in frequency (Fig. 4B). While the desynchronization of frequencies below 5 Hz was short in time and lasted up to 8 s in the ACC, that of frequencies between 6 and 20 Hz was strong and extended 16 s post-stimulation onset in S1HL and EEG, and 8 s in ACC.

The observed decrease in the power between 6 and 20 Hz in NREMS (Fig. 4B) may be driven by the termination of sleep spindles (11–16 Hz) upon awakening. Therefore, we evaluated whether these oscillatory changes were also present in the absence of awakening (Fig. 4C). The desynchronization in the frequency range between 6 and 20 Hz was maintained in S1HL, weakened and prolonged in ACC, and narrowed to 6–16 Hz in EEG. Thus, the decrease in the sleep spindle range was not linked to awakening and may reflect decreased sleep quality. The temporal biphasic response of gamma was also preserved, although the power increases were of different amplitude and durations, suggesting that bottom-up and top-down mechanisms were differentially engaged. In the absence of awakening, a new response, consisting of an increase in the power of slow frequencies (<6 Hz), emerged.

**Fig. 5:**
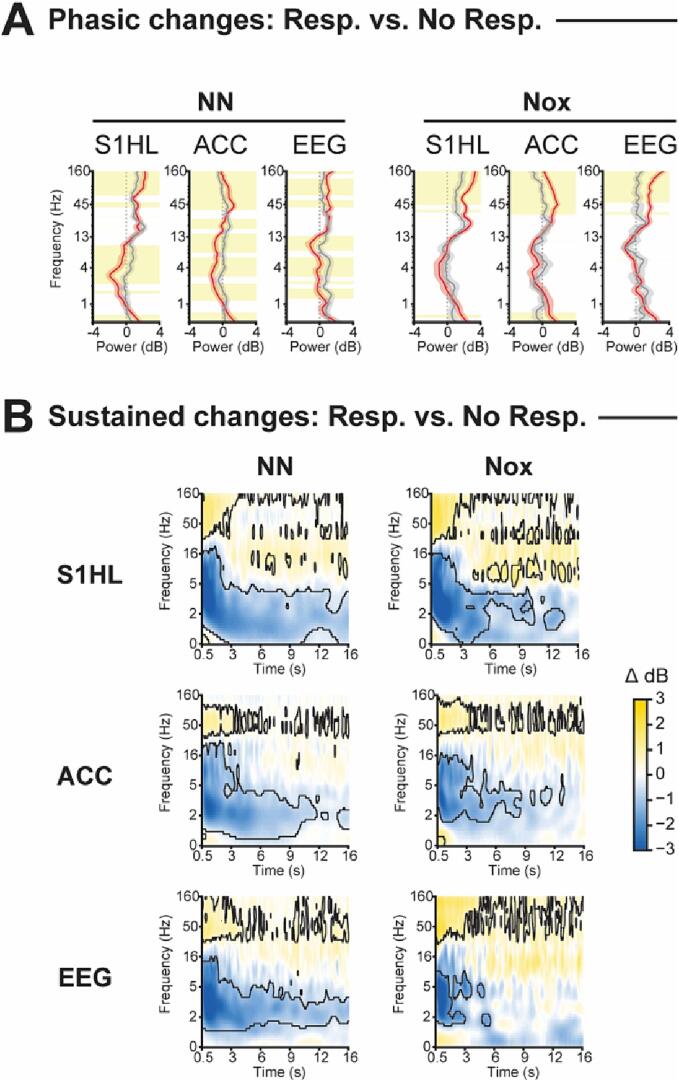
Comparison of the spectral properties during NREMS of stimuli evoking awakening vs. stimuli without awakening. A, Spectral profiles of the phasic response (0 – 400 msec post-stimulation onset) in dB. Vertical dotted line at zero defines no change from baseline. Data were represented as mean ± SEM. Yellow shaded areas indicate statistically significant differences at p < 0.05, FDR corrected. B, Differential sustained response. Black contour lines delineate statistically significant differences between response and no response (p < 0.05, FDR corrected), N = 17. (For interpretation of the references to colour in this figure legend, the reader is referred to the web version of this article.)

Even though the general pattern of stimulus-induced changes in the spectro-temporal profile was maintained in the absence of awakening, it showed differences to that including all behavioral responses. Thus, we compared the spectral properties of stimuli with and without awakenings (Fig. 5). We defined an early window of 400 msec right after stimulation onset to compare the phasic bottom-up processes (Fig. 5A). Then, using a larger window between 0.5 and 16 s, we computed the differential spectrograms of stimuli with and without awakening to characterize the differences of the sustained top-down mechanisms (Fig. 5B). Awakening evoked an initial significant increase of broadband gamma (>30 Hz) across conditions and recording sites (Fig. 5A). This increase was maintained, as observed in the sustained response, where it kept its broadband characteristics in S1HL and EEG but became narrowband in the ACC (Fig. 5B). Awakening from non-noxious stimuli was additionally associated with phasic decreases of frequencies below 16 Hz (Fig. 5A), with spectral properties that were specific for each recording site and that extended into the sustained response, prolonging up to 16 s post-stimulation onset (Fig. 5B, left column). Awakenings induced by noxious stimuli did not significantly decrease the power in the frequencies below 16 Hz in the phasic response compared to non-awakenings (Fig. 5A). The significant decrease appeared later, in the sustained response (Fig. 5B, right column), although the duration of which was shorter compared to non-noxious stimuli. In summary, the power decrease in frequencies below 16 Hz in the sustained response was larger for those stimuli with awakening compared to those without. This effect was more pronounced and long-lasting in non-noxious compared to noxious stimulation. Thus, stimulus-induced-wake-like features were more prominent in noxious than in non-noxious stimuli in the absence of awakening.

In wake, previous reports have observed differences in gamma activity in S1 between response and no-response (Tan et al., 2019). Therefore, we analyzed the phasic and sustained responses of stimuli applied during wake (Suppl. Fig. 8). We found no differences between response and no-response neither in the phasic window of stimuli applied in wake nor in any sustained response window. The discrepancy of our results with previous studies may be explained by the different states of wake included in the study. While previous reports applied stimuli during “quiet wake” (Tan et al., 2019), we applied the stimuli when the animal had all four paws on the grid, thus, generating a heterogeneous representation of wake. We additionally detected that non-noxious stimuli evoked significantly greater decreases of frequencies below 2 Hz in the ACC 6 s after stimulation (Suppl. Fig. 5B).

Also in wake, larger early gamma increases have been described for noxious compared to non-noxious stimulation (Zhang et al., 2012). Therefore, we compared the spectro-temporal profiles of both stimulus types (Suppl. Fig. 9). We found that in NREMS, but not wake, phasic gamma increases to noxious stimuli were significantly larger than to non-noxious stimuli, but only if they induced an awakening (Suppl. Fig. 9A). Noxious stimuli additionally evoked pronounced increases in frequencies above 16 Hz in S1HL and ACC, although in ACC not all frequencies above 16 Hz were significantly boosted. In the EEG, significant noxious-stimulation-induced power increases were restricted to frequencies over 80 Hz. In S1HL, non-noxious stimulation during NREM additionally evoked larger decreases of frequencies between 1 and 4 Hz and less pronounced increases in frequencies below 1 Hz in the phasic window that did not extend to the sustained response window. However, the sustained responses in NREM showed that noxious stimulation caused larger decreases at 12–18 Hz in S1HL and 5–16 Hz in ACC extending up to 16 s. This effect was not present in the EEG. Thus, the localized decrease of the sleep spindle range was stronger upon noxious than non-noxious stimulation.

In summary, our findings indicate that while the responses to somatic stimuli vary between wakefulness and NREMS, an increase in gamma power occurs in both states, even without awakening. This is a further indication that somatosensory processing occurs in cortical areas during NREMS. Nevertheless, the most significant alteration in the recorded oscillatory patterns is linked to the presence of a behavioral response, whether in wakefulness or NREMS. Finally, we found that both noxious and non-noxious stimuli produce similar spectro-temporal changes, implying similar activation in both brain areas regardless of the stimulation type.

## Heart rate correlations with local oscillatory activity in the gamma range distinguishes noxious from non-noxious stimulation in wake and NREMS

**Fig. 6:**
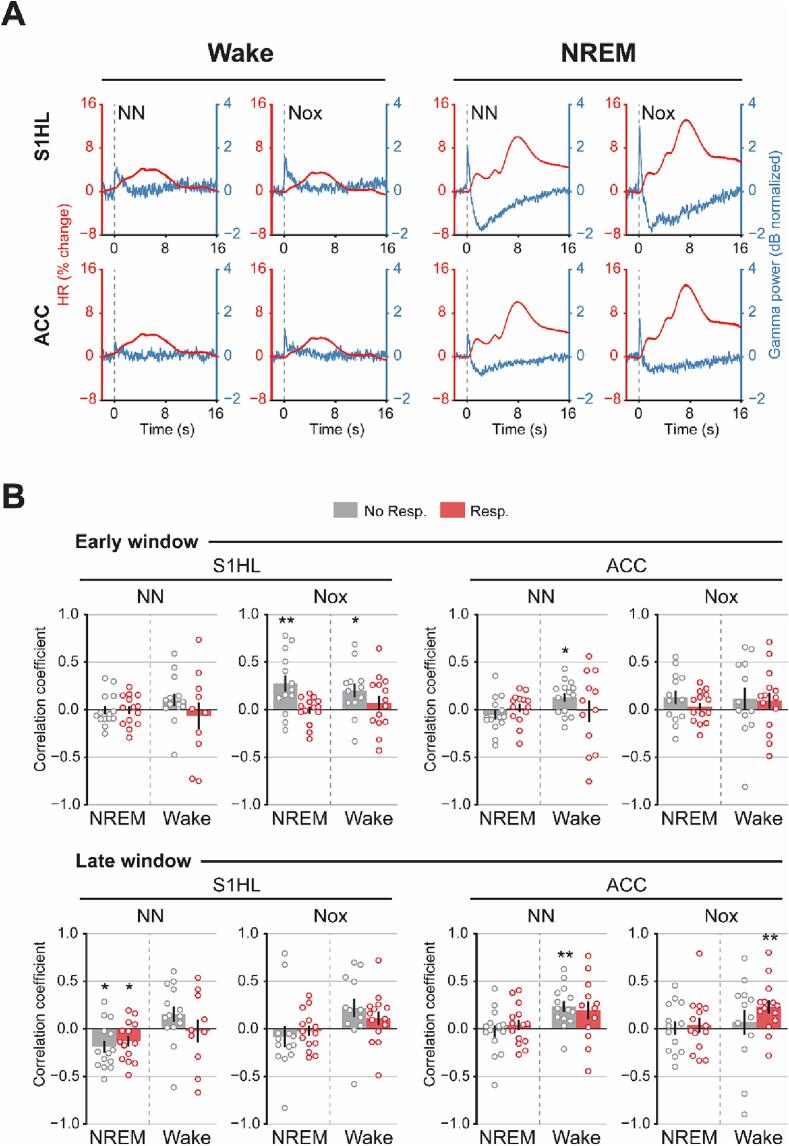
Heart rate single-trial correlations with gamma frequency band. A, Time series of heart rate and gamma power represented. Line plots represented as the mean. B, Single-trial correlations between heart rate and power in gamma band. Each data point represents one animal. Data were represented as mean ± SEM. *, p < 0.05, **, p < 0.01. N = 14.

Despite the finding that both noxious and non-noxious stimuli evoked similar spectro-temporal profiles (Fig. 4), the increase in gamma power was larger for noxious as compared to non-noxious stimulation in the presence of a behavioral response during NREMS (Suppl. Fig. 9). Interestingly, increases in heart rate were larger for noxious than for non-noxious stimulation across conditions (Fig. 2). The overlay of the baseline normalized heart rate and the gamma power (Fig. 6A) indicated that sensory stimulation co-modulated these two measures. As both gamma power and heart rate increase with rising pain intensity (Heid et al., 2020, Lechner et al., 1998, Zhang et al., 2012), and brain rhythms might be influenced by the heartbeat directly (Al et al., 2020), we performed single-trial correlations of the unprocessed signals of the heart rate and local gamma activity for ACC and S1HL contralateral to the stimulated paw. These correlations were computed for an early and a late time window to separate the phasic from the sustained effect of the stimulation, respectively.

Single-trial correlations in the early window showed that only noxious stimuli that did not evoke a behavioral response resulted in positive gamma-heart rate correlations in S1HL in both wake (0.20 ± 0.07, T(11) = 2.5, p = 0.02) and NREMS (0.27 ± 0.08, T(12) = 3.08, p = 0.009) (Fig. 6B, top row). In ACC, only non-noxious stimuli in wake in the absence of overt behavioral responses resulted in a positive correlation in the early window (0.12 ± 0.04, T(11) = 2.6, p = 0.019). These results suggest that S1HL cortical gamma activity conveys information about noxious sensory inputs and are not simply due to muscle activation or saliency. The lack of pattern of correlations in ACC does not allow for any specific conclusion.

In the late window, non-noxious stimulation evoked negative gamma-heart rate correlations during NREMS in S1HL, independently of the behavioral response (NoResp: −0.18 ± 0.06, T(13) = -2.8, p = 0.013, Resp: −0.13 ± 0.05, T(13) = -2.3, p = 0.03) (Fig. 6B, bottom row), that were likely driven by a larger gamma power decrease after the initial phasic increase in NREMS compared to wake (Fig. 6A). Despite noxious stimulation evoking similar temporal dynamics in S1HL (Fig. 6A), no correlation was found (Fig. 6B, bottom row), likely due to a larger variability for noxious compared to non-noxious stimulation. In ACC, during wake, non-noxious stimulation evoked significantly positive correlations in the absence of a behavioral response (0.23 ± 0.05, T(13) = 4.08, p = 0.001). Noxious stimulation also evoked a significantly positive correlation only if a motor response was induced (0.23 ± 0.07, T(13) = 3.2, p = 0.006). Nevertheless, no evident pattern was present in the late window to distinguish noxious from non-noxious stimulation. Thus, the correlations that allow to separate both types of stimuli are specific to an early time window.

In summary, single-trial correlations of the phasic responses of heart rate and gamma power might allow to distinguish between nociceptive – pain-related, such as pinprick – from exclusively salient – non-pain related, such as touch – somatic stimuli in different states of consciousness (wake and NREMS) when a behavioral response is not present.

## Discussion

In this study we showed that somatic stimuli activated the autonomic nervous system across arousal states (Fig. 2). Then, using neural data from intracranial recordings, we showed that peripheral somatic information reached cortical areas involved in the processing of the sensory (S1) and affective (ACC) components of pain during different arousal states (Fig. 3). Analysis of the stimulus-evoked spectro-temporal dynamics provided a characterization of noxious and non-noxious processing in S1HL and ACC, confirming the processing of different somatic stimuli during NREMS (Fig. 4). Lastly, because pain differentially co-modulates brain and autonomic activity, we correlated neural activity with heart rate demonstrating that nociception can be distinguished from saliency when using multiple electrophysiological measures (Fig. 5).

### The biological saliency of the stimulation likely influences the computational flow and the sensory filtering characteristic of sleep

Our analysis of SSEPs (Fig. 2) allowed us to evaluate the neural signatures of somatic stimuli. In wake, somatosensory information first reached S1HL and then, ACC, which is consistent with the spatio-temporal structure of information processing shown in other studies (Anderson et al., 2017, Le Merre et al., 2018, Leong et al., 2016). This effect was also observed in NREMS, suggesting that the computational flow – the cortical recruitment driven by somatic information – is preserved during sleep. The differential engagement of local neural circuits (last peak) by both stimuli suggests that the nuances of somatic stimuli are discernible in S1HL during wakefulness, while, during NREMS, this differentiation is possible in both cortical areas. Notably, the advancement of the second peak in NREMS by noxious stimulation suggests a quicker engagement of local feed-back circuits, possibly prioritizing highly salient stimuli, given that the generation of an adequate behavioral response during NREMS may be crucial for survival. Non-noxious stimulation during NREMS recruited local S1HL circuits and thalamo-cortical bottom-up processes in ACC later than in wake. Moreover, the amplitude of the first peak in S1 was lower in NREMS compared to wake only upon non-noxious stimulation, suggesting that low-saliency/non-nociceptive somatosensory stimulation undergoes sensory filtering during sleep, while highly salient/nociceptive information, does not.

All these results taken together, suggest that sensory attenuation during sleep is based on the biological relevance, i.e. saliency, of the stimulation. Hence, low-saliency stimuli, such as non-noxious stimulation, would be attenuated as described in the thalamic gating hypothesis (Andrillon and Kouider, 2020, McCormick and Bal, 1994, Steriade, 2003). On the other hand, highly salient stimuli, such as noxious stimulation, would be prioritized, by engaging cortical areas faster and by avoiding sensory filtering, given that a timely response can be crucial for survival. This prioritization effect may partially explain the increased rates of awakening caused by salient stimuli such as nociceptive stimulation (Fig. 1G) (Bentley et al., 2003) or hearing one’s own name (Perrin et al., 1999).

### Somatic stimuli increase awareness to the surroundings in the absence of awakening

Both noxious and non-noxious stimuli decreased the power of sigma, the frequency range corresponding to sleep spindles (11–16 Hz) (Fig. 4B,C). Sleep spindles are a hallmark of NREMS, protect sleep from environmental disturbances and are terminated upon awakening (Fernandez & Lüthi, 2020). In the absence of a behavioral response, our results showed a stimulus-evoked reduction of sleep spindles that was stronger in S1HL than in ACC (Fig. 4C), suggesting that the effect of the stimulation on sleep spindles is brain region specific. This reduction is likely driven by the release of norepinephrine by the locus coeruleus (LC) to the thalamus (Fernandez & Lüthi, 2020). LC has been found to become active throughout the sleep cycle (Osorio-Forero et al., 2021), which has been interpreted as a mechanism to increase environmental awareness to scan for potential threads during sleep (Osorio-Forero et al., 2021, Osorio-Forero et al., 2022). Thus, we posit that a stimulus-induced decrease in sigma may be indicative of heightened vigilance during NREMS and a reduced gating of peripheral sensory information to cortical areas. This effect would be a priming mechanism to rapidly generate an adequate response ensuing potentially threatening stimuli. A second stimulation within the 16 s after the onset of the stimulation would allow testing this hypothesis by evaluating changes in the rate of awakening.

Nevertheless, the long-lasting effect of the stimulation on the sigma range is a considerable disruption of NREMS. Hence, salient stimuli during sleep likely disturb sleep quality despite the absence of a behavioral correlate. It would be interesting to link this phenomenon with subsequent behaviors during wake such as memory, learning or attention given that sleep spindles are implicated in these phenomena (Egawa et al., 2021, Fernandez and Lüthi, 2020, Lustenberger et al., 2012, Peyrache and Seibt, 2020).

### Correlation of neural oscillatory activity with heart rate measures has potential to disentangle processes sharing similar spectral characteristics

In our hands, noxious and non-noxious stimulation evoked very similar spectro-temporal profiles both in wake and in NREMS (Fig. 3). Thus, these results could argue in favor of the hypothesis that the resulting oscillatory changes by a short stimulation, such as the ones we delivered to the animals (Fig. 1D), are rather the effect of saliency instead of somatosensation (Iannetti and Mouraux, 2010, Legrain et al., 2011). Indeed, the spectral profile of saliency and sensory processing are highly intertwined (Iannetti and Mouraux, 2010, Legrain et al., 2011). In addition, noxious stimulation activates a vast number of brain areas (Apkarian et al., 2005, Da Silva and Seminowicz, 2019, Garcia-Larrea and Peyron, 2013, Peyron et al., 1999), many of which belong to the saliency network (De Ridder et al., 2022). However, transient noxious stimulation evokes changes not only in brain areas but also in heart rate (Fig. 2) (Forte et al., 2022a, Forte et al., 2022b, Hilgard et al., 1974, Loggia et al., 2011, Möltner et al., 1990, Terkelsen et al., 2005, Tousignant-Laflamme et al., 2005) and therefore, combining neural and bodily physiological reactions to stimuli has the potential to better interpret neural activity. Here, we showed that in the absence of overt behavioral responses, the correlations between heart rate and gamma power in S1HL can distinguish noxious from non-noxious stimulation better than either of the two measurements alone, during wake and NREMS. Furthermore, this effect is brain region dependent, given that the same pattern of correlations did not appear in ACC (Fig. 6B). These results indicate that heart rate-S1 gamma correlations have the potential to isolate the saliency from the sensory component. Thus, this type of correlation holds great potential as a tool to identify nociception in the absence of a behavioral report, such as in the case of animals, human babies, sleep or comatose patients.

### Limitations of the study

It is important to acknowledge that the current study presents some limitations. First, we exclusively used male mice and differences in the processing and perception of pain between genders have been documented. Human studies have revealed that women report greater sensitivity to multiple acute pain modalities (Bartley and Fillingim, 2013, Wiesenfeld-Hallin, 2005) that are mediated by psychosocial and biological mechanisms (Bartley and Fillingim, 2013, Dance, 2019, Wiesenfeld-Hallin, 2005). In fact, EEG recordings showed greater amplitudes of laser evoked potentials in women than in men (Staikou et al., 2017). Furthermore, sleep is more fragile in females than in men in both humans (Jonasdottir et al., 2021, Mong and Cusmano, 2016, Zendels et al., 2021) and rodents (Choi et al., 2021, Dib et al., 2021). Therefore, an important avenue for pain research includes the investigation of sex differences in pain processing during sleep.

Second, we have argued that correlating heart rate with S1 gamma activity can aid to disentangle the saliency from the sensory component. However, both stimuli are of the same modality and therefore, the use of other sensory modalities, such as auditory or visual stimuli, would be of great advantage to further inform how to differentiate saliency- from nociceptive-related neural activity.

In summary, here we have shown that somatic stimuli reach the cortex during NREMS in a similar fashion as in wake and that noxious can be distinguished from non-noxious stimulation during NREMS. Our results also show that the spectro-temporal dynamics evoked by these two types of stimuli present oscillatory changes that are related to information processing and attention in both wake and NREMS. Furthermore, correlating oscillatory neural activity with heart rate measurements has a great potential to distinguish nociception from touch in the absence of a behavioral response.

### CRediT authorship contribution statement

**Sandoval Ortega Raquel Adaia:** Conceptualization, Performed research, Data collection, Data curation, Sleep scoring, Data analysis, Software, Validation, Methodology, Figures, Writing – original draft, Writing – review & editing. **Renard Margot:** Performed research, Data collection, Data curation, Sleep scoring, Writing – review & editing. **Cohen Michael X.:** Supervision, Analysis, Writing – review & editing. **Nevian Thomas:** Conceptualization, Funding acquisition, Supervision, Writing – review & editing.

## Declaration of competing interest

The authors declare the following financial interests/personal relationships which may be considered as potential competing interests: This work was supported by the Swiss National Science Foundation (T.N., grant 182571) and the European Research Council (T.N., grant 682905).

## Data Availability

Data will be made available on request.

## References

[b0005] Acuña M.A., Kasanetz F., De Luna P., Falkowska M., Nevian T. (2023). Principles of nociceptive coding in the anterior cingulate cortex. PNAS.

[b0010] Agarwal N., Choi P.A., Shin S.S., Hansberry D.R., Mammis A. (2016). Anterior cingulotomy for intractable pain. Interdisciplinary Neurosurgery.

[b0015] Al E., Iliopoulos F., Forschack N., Nierhaus T., Grund M., Motyka P., Gaebler M., Nikulin V.V., Villringer A. (2020). Heart-brain interactions shape somatosensory perception and evoked potentials. PNAS.

[b0020] Anderson P.M., Jones N.C., O’Brien T.J., Pinault D. (2017). The N -Methyl d -aspartate glutamate receptor antagonist ketamine disrupts the functional state of the corticothalamic pathway. Cereb. Cortex.

[b0025] Andrillon T., Kouider S. (2020). The vigilant sleeper: neural mechanisms of sensory (de)coupling during sleep. Curr. Opinion Physiol..

[b0030] Andrillon T., Poulsen A.T., Hansen L.K., Léger D., Kouider S. (2016). Neural Markers of Responsiveness to the Environment in Human Sleep. J. Neurosci..

[b0035] Apkarian A.V., Bushnell M.C., Treede R.D., Zubieta J.K. (2005). Human brain mechanisms of pain perception and regulation in health and disease. Eur. J. Pain.

[b0040] Bartley E.J., Fillingim R.B. (2013). Sex differences in pain: a brief review of clinical and experimental findings. BJA: British Journal of Anaesthesia.

[b0045] Başar E., Başar-Eroglu C., Karakaş S., Schürmann M. (1999). Are cognitive processes manifested in event-related gamma, alpha, theta and delta oscillations in the EEG?. Neurosci. Lett..

[b0050] Başar-Eroglu C., Strüber D., Schürmann M., Stadler M., Başar E. (1996). Gamma-band responses in the brain: a short review of psychophysiological correlates and functional significance. Int. J. Psychophysiol..

[b0055] Bastuji H., Mazza S., Perchet C., Frot M., Mauguière F., Magnin M., Garcia-Larrea L. (2012). Filtering the reality: Functional dissociation of lateral and medial pain systems during sleep in humans. Hum. Brain Mapp..

[b0060] Bentley A.J., Newton S., Zio C.D. (2003). Sensitivity of sleep stages to painful thermal stimuli. J. Sleep Res..

[b0065] Buzsáki, G., Anastassiou, C. A., & Koch, C. (2012). The origin of extracellular fields and currents — EEG, ECoG, LFP and spikes. *Nature Reviews Neuroscience 2012 13:6*, *13*(6), 407–420. https://doi.org/10.1038/nrn3241. 10.1038/nrn3241 PMC4907333 22595786

[b0070] Buzsáki G., Logothetis N., Singer W. (2013). Scaling brain size, keeping timing: evolutionary preservation of brain rhythms. Neuron.

[b0075] Choi J., Kim S.J., Fujiyama T., Miyoshi C., Park M., Suzuki-Abe H., Yanagisawa M., Funato H. (2021). The role of reproductive hormones in sex differences in sleep homeostasis and arousal response in mice. Front. Neurosci..

[b0080] Chouchou F., Pichot V., Perchet C., Legrain V., Garcia-Larrea L., Roche F., Bastuji H. (2011). Autonomic pain responses during sleep: a study of heart rate variability. Eur. J. Pain (london, England).

[b0085] Cohen M.X. (2014). Analyzing neural time series data: theory and practice. Analyzing Neural Time Series Data.

[b0090] Cohen M.X. (2017). Where does EEG come from and what does it mean?. Trends Neurosci..

[b0095] Da Silva J.T., Seminowicz D.A. (2019). Neuroimaging of pain in animal models: a review of recent literature. Pain Reports.

[b0100] Dance A. (2019). Why the sexes don’t feel pain the same way. Nature.

[b0105] De Ridder D., Vanneste S., Smith M., Adhia D. (2022). Pain and the triple network model. Front. Neurol..

[b0110] Dib R., Gervais N.J., Mongrain V. (2021). A review of the current state of knowledge on sex differences in sleep and circadian phenotypes in rodents. Neurobiol. Sleep Circadian Rhythms.

[b0115] Egawa K., Saitoh S., Asahina N., Shiraishi H. (2021). Short-latency somatosensory-evoked potentials demonstrate cortical dysfunction in patients with Angelman syndrome. ENeurologicalSci.

[b0120] Fernandez L.M.J., Lüthi A. (2020). Sleep spindles: Mechanisms and functions. Physiol. Rev..

[b0125] Finan P.H., Goodin B.R., Smith M.T. (2013). The association of sleep and pain: an update and a path forward. J. Pain.

[b0130] Forte G., Troisi G., Pazzaglia M., De Pascalis V., Casagrande M. (2022). Heart rate variability and pain. A Systematic Review. *Brain Sciences*.

[b0135] Forte G., Troisi G., Pazzaglia M., Pascalis V.D., Casagrande M. (2022). Heart rate variability and pain: a systematic review. Brain Sci..

[b0140] Fransen A.M.M., Dimitriadis G., van Ede F., Maris E. (2016). Distinct α- and β-band rhythms over rat somatosensory cortex with similar properties as in humans. J. Neurophysiol..

[b0145] Garcia-Larrea L., Peyron R. (2013). Pain matrices and neuropathic pain matrices: a review. Pain.

[b0150] Goldberger J.J., Challapalli S., Tung R., Parker M.A., Kadish A.H. (2001). Relationship of heart rate variability to parasympathetic effect. Circulation.

[b0155] Hauck M., Domnick C., Lorenz J., Gerloff C., Engel A.K. (2015). Top-down and bottom-up modulation of pain-induced oscillations. Front. Hum. Neurosci..

[b0160] Heid C., Mouraux A., Treede R.D., Schuh-Hofer S., Rupp A., Baumgärtner U. (2020). Early gamma-oscillations as correlate of localized nociceptive processing in primary sensorimotor cortex. J. Neurophysiol..

[b0165] Herreras O. (2016). Local field potentials: Myths and misunderstandings. Front. Neural Circuits.

[b0170] Hilgard E.R., Morgan A.H., Lange A.F., Lenox J.R., Macdonald H., Marshall G.D., Sachs L.B. (1974). Heart rate changes in pain and hypnosis. Psychophysiology.

[b0175] Iannetti, G. D., & Mouraux, A. (2010). From the neuromatrix to the pain matrix (and back). *Experimental Brain Research 2010 205:1*, *205*(1), 1–12. https://doi.org/10.1007/S00221-010-2340-1. 10.1007/s00221-010-2340-1 20607220

[b0180] Ikkai A., Dandekar S., Curtis C.E. (2016). Lateralization in alpha-band oscillations predicts the locus and spatial distribution of attention. PLoS One.

[b0185] Jonasdottir S.S., Minor K., Lehmann S. (2021). Gender differences in nighttime sleep patterns and variability across the adult lifespan: a global-scale wearables study. Sleep.

[b0190] Kakigi R., Naka D., Okusa T., Wang X., Inui K., Qiu Y., Tran T.D., Miki K., Tamura Y., Nguyen T.B., Watanabe S., Hoshiyama M. (2003). Sensory perception during sleep in humans: a magnetoencephalograhic study. Sleep Med..

[b0195] Karakaş S., Başar-Eroǧlu C., Özesmi Ç., Kafadar H., Erzengin Ö.Ü. (2001). Gamma response of the brain: a multifunctional oscillation that represents bottom-up with top-down processing. Int. J. Psychophysiol..

[b0200] Kasanetz, F., Acuña, M.A., Nevian, T. (2021) Anterior cingulate cortex, pain perception, and pathological neuronal plasticity during chronic pain. The Neurobiology, Physiology, and Psychology of Pain, pp. 193-202. Elsevier. doi: 10.1016/B978-0-12-820589-1.00018-X.

[b0205] Kasanetz F., Nevian T. (2021). Increased burst coding in deep layers of the ventral anterior cingulate cortex during neuropathic pain. Sci. Rep..

[b0210] Kazmi S.Z.H., Zhang H., Aziz W., Monfredi O., Abbas S.A., Shah S.A., Kazmi S.S.H., Butt W.H. (2016). Inverse Correlation between Heart Rate Variability and Heart Rate Demonstrated by Linear and Nonlinear Analysis. PLoS One.

[b0215] Kitamura Y., Kakigi R., Hoshiyama M., Koyama S., Nakamura A. (1996). Effects of sleep on somatosensory evoked responses in human: a magnetoencephalographic study. Brain Res. Cogn. Brain Res..

[b0220] Koenig J., Jarczok M.N., Ellis R.J., Hillecke T.K., Thayer J.F. (2014). Heart rate variability and experimentally induced pain in healthy adults: A systematic review. European Journal of Pain (united Kingdom).

[b0225] Le Merre P., Esmaeili V., Charrière E., Galan K., Salin P.A., Petersen C.C.H., Crochet S. (2018). Reward-Based Learning Drives Rapid Sensory Signals in Medial Prefrontal Cortex and Dorsal Hippocampus Necessary for Goal-Directed Behavior. Neuron.

[b0230] Lechner D.E., Bradbury S.F., Bradley L.A. (1998). Detecting sincerity of effort: a summary of methods and approaches. Phys. Ther..

[b0235] Legrain V., Iannetti G.D., Plaghki L., Mouraux A. (2011). The pain matrix reloaded: A salience detection system for the body. Prog. Neurobiol..

[b0240] Leong, A. T. L., Chan, R. W., Gao, P. P., Chan, Y. S., Tsia, K. K., Yung, W. H., & Wu, E. X. (2016). Long-range projections coordinate distributed brain-wide neural activity with a specific spatiotemporal profile. *Proceedings of the National Academy of Sciences of the United States of America*, *113*(51), E8306–E8315. https://doi.org/10.1073/PNAS.1616361113/SUPPL_FILE/PNAS.201616361SI.PDF. 10.1073/pnas.1616361113 PMC5187697 27930323

[b0245] Loggia M.L., Juneau M., Bushnell M.C. (2011). Autonomic responses to heat pain: Heart rate, skin conductance, and their relation to verbal ratings and stimulus intensity. Pain.

[b0250] Lustenberger C., Maric A., Dürr R., Achermann P., Huber R. (2012). Triangular relationship between sleep spindle activity, general cognitive ability and the efficiency of declarative learning. PLoS One.

[b0255] Magosso E., De Crescenzio F., Ricci G., Piastra S., Ursino M. (2019). EEG alpha power is modulated by attentional changes during cognitive tasks and virtual reality immersion. Comput. Intell. Neurosci..

[b0260] Mazza S., Magnin M., Bastuji H. (2012). Pain and sleep: From reaction to action. Neurophysiologie Clinique/clinical Neurophysiology.

[b0265] McCormick D.A., Bal T. (1994). Sensory gating mechanisms of the thalamus. Curr. Opin. Neurobiol..

[b0270] Möltner A., Hölzl R., Strian F. (1990). Heart rate changes as an autonomic component of the pain response. Pain.

[b0275] Mong J.A., Cusmano D.M. (2016). Sex differences in sleep: impact of biological sex and sex steroids. Philos. Trans. R. Soc., B.

[b0280] Nir, Y., Vyazovskiy, V. V., Cirelli, C., Banks, M. I., & Tononi, G. (2015). Auditory responses and stimulus-specific adaptation in rat auditory cortex are preserved across NREM and REM sleep. *Cerebral Cortex (New York, N.Y. : 1991)*, *25*(5), 1362–1378. https://doi.org/10.1093/CERCOR/BHT328. 10.1093/cercor/bht328 PMC4415088 24323498

[b0285] Osorio-Forero A., Cardis R., Vantomme G., Guillaume-Gentil A., Katsioudi G., Devenoges C., Fernandez L.M.J., Lüthi A. (2021). Noradrenergic circuit control of non-REM sleep substates. Curr. Biol..

[b0290] Osorio-Forero A., Cherrad N., Banterle L., Fernandez L.M.J., Lüthi A. (2022). When the locus coeruleus speaks up in sleep: recent insights, emerging perspectives. Int. J. Mol. Sci..

[b0295] Perrin F., García-Larrea L., Mauguière F., Bastuji H. (1999). A differential brain response to the subject’s own name persists during sleep. Clin. Neurophysiol..

[b0300] Peyrache A., Seibt J. (2020). A mechanism for learning with sleep spindles. Philos. Trans. R. Soc. B.

[b0305] Peyron R., García-Larrea L., Grégoire M.C., Costes N., Convers P., Lavenne F., Mauguière F., Michel D., Laurent B. (1999). Haemodynamic brain responses to acute pain in humansSensory and attentional networks. Brain.

[b0310] Sacha, J. (2014). Interaction between Heart Rate and Heart Rate Variability. *Annals of Noninvasive Electrocardiology : The Official Journal of the International Society for Holter and Noninvasive Electrocardiology, Inc*, *19*(3), 207. https://doi.org/10.1111/ANEC.12148. 10.1111/anec.12148 PMC6932101 24602150

[b0315] Sela Y., Krom A.J., Bergman L., Regev N., Nir Y. (2020). Sleep differentially affects early and late neuronal responses to sounds in auditory and perirhinal cortices. J. Neurosci..

[b0320] Sharon O., Nir Y. (2018). Attenuated fast steady-state visual evoked potentials during human sleep. Cereb. Cortex.

[b0325] Shaw F.Z., Lee S.Y., Chiu T.H. (2006). Modulation of somatosensory evoked potentials during wake-sleep states and spike-wave discharges in the rat. Sleep.

[b0330] Sobolewski A., Swiejkowski D.A., Wróbel A., Kublik E. (2011). The 5–12 Hz oscillations in the barrel cortex of awake rats – Sustained attention during behavioral idling?. Clin. Neurophysiol..

[b0335] Staikou C., Kokotis P., Kyrozis A., Rallis D., Makrydakis G., Manoli D., Karandreas N., Stamboulis E., Moschovos C., Fassoulaki A. (2017). Differences in pain perception between men and women of reproductive age: a laser-evoked potentials study. Pain Med..

[b0340] Steriade M. (2003). The corticothalamic system in sleep. Front Biosci.

[b0345] Su, Q., Song, Y., Zhao, R., & Liang, M. (2020). A review on the ongoing quest for a pain signature in the human brain. *Https://Doi.Org/10.26599/BSA.2019.9050024*, *5*(4), 274–287. https://doi.org/10.26599/BSA.2019.9050024.

[b0350] Tan L.L., Oswald M.J., Heinl C., Retana Romero O.A., Kaushalya S.K., Monyer H., Kuner R. (2019). Gamma oscillations in somatosensory cortex recruit prefrontal and descending serotonergic pathways in aversion and nociception. Nat. Commun..

[b0355] Terkelsen A.J., Mølgaard H., Hansen J., Andersen O.K., Jensen T.S. (2005). Acute pain increases heart rate: differential mechanisms during rest and mental stress. Auton. Neurosci..

[b0360] Thorpe, R. V., Black, C. J., Borton, D. A., Hu, L., Saab, C. Y., & Jones, S. R. (2021). Distinct neocortical mechanisms underlie human SI responses to median nerve and laser evoked peripheral activation. *BioRxiv*, 2021.10.11.463545. https://doi.org/10.1101/2021.10.11.463545. 10.1162/imag_a_00095 PMC12235563 40800401

[b0365] Tousignant-Laflamme Y., Rainville P., Marchand S. (2005). Establishing a link between heart rate and pain in healthy subjects: a gender effect. J. Pain.

[b0370] Wang X., Inui K., Qiu Y., Kakigi R. (2004). Cortical responses to noxious stimuli during sleep. Neuroscience.

[b0375] Wiesenfeld-Hallin Z. (2005). Sex differences in pain perception. Gend. Med..

[b0380] Woodman G.F. (2010). A brief introduction to the use of event-related potentials (ERPs) in studies of perception and attention. Atten. Percept. Psychophys..

[b0385] Wöstmann M., Maess B., Obleser J. (2021). Orienting auditory attention in time: Lateralized alpha power reflects spatio-temporal filtering. Neuroimage.

[b0390] Zendels P., Ruggiero A., Gaultney J.F. (2021). Gender differences affecting the relationship between sleep attitudes, sleep behaviors and sleep outcomes. Http://www.editorialmanager.com/cogentpsychology.

[b0395] Zhang Z.G., Hu L., Hung Y.S., Mouraux A., Iannetti G.D. (2012). Gamma-band oscillations in the primary somatosensory cortex—a direct and obligatory correlate of subjective pain intensity. J. Neurosci..

