# Supplementary material for: Interactive effects of pain and arousal state on heart rate and cortical activity in the mouse anterior cingulate and somatosensory cortices
Source: Neurobiol Pain. 2024 Apr 25;15:100157. doi: 10.1016/j.ynpai.2024.100157 (PMC11099324; doi:10.1016/j.ynpai.2024.100157)
Supplement: Supplementary Data 1 [file mmc1.pdf]

Interactive Effects of Pain and Arousal State on Heart Rate and Cortical Activity in the Mouse Anterior Cingulate and Somatosensory Cortices

Sandoval Ortega, Raquel Adaia [1], Renard, Margot [1], Cohen, Michael X [2], Nevian, Thomas [1]

Supplementary Tables

	F	p
Stim. Type	0.03	0.8
Arousal	20.08	0.4×10^{-4} **
Stim. Type : Arousal	1.08	0.3

Supplementary Table 1. Results of a 2-way ANOVA to assess the effects of Stimulation type and Arousal and their interactions on HRV. Statistics (F) and p-values (p) for HRV corresponding to Supplementary Figure 5 A and B. Calculated from the mean of an 8-second window (see methods). *: $p < 0.05$, **: $p < 0.01$.

	NN			Nox	
	F	p		F	p
Arousal	30.60	1×10^{-6} **		13.91	4.74×10^{-4} **
Behav. Resp	5.30	0.025 *		1.64	0.206
Arousal : Behav. Resp	3.33	0.074		2.68	0.107

Supplementary Table 2. Results of a 2-way ANOVA on HRV to assess the factors Arousal and Behavioral Response after non-noxious (NN) and noxious (Nox) stimulation. Statistics (F) and p-values (p) for HRV of an 8 seconds window corresponding to Figure 2C and D. *: $p < 0.05$, **: $p < 0.01$.

Supplementary Figures

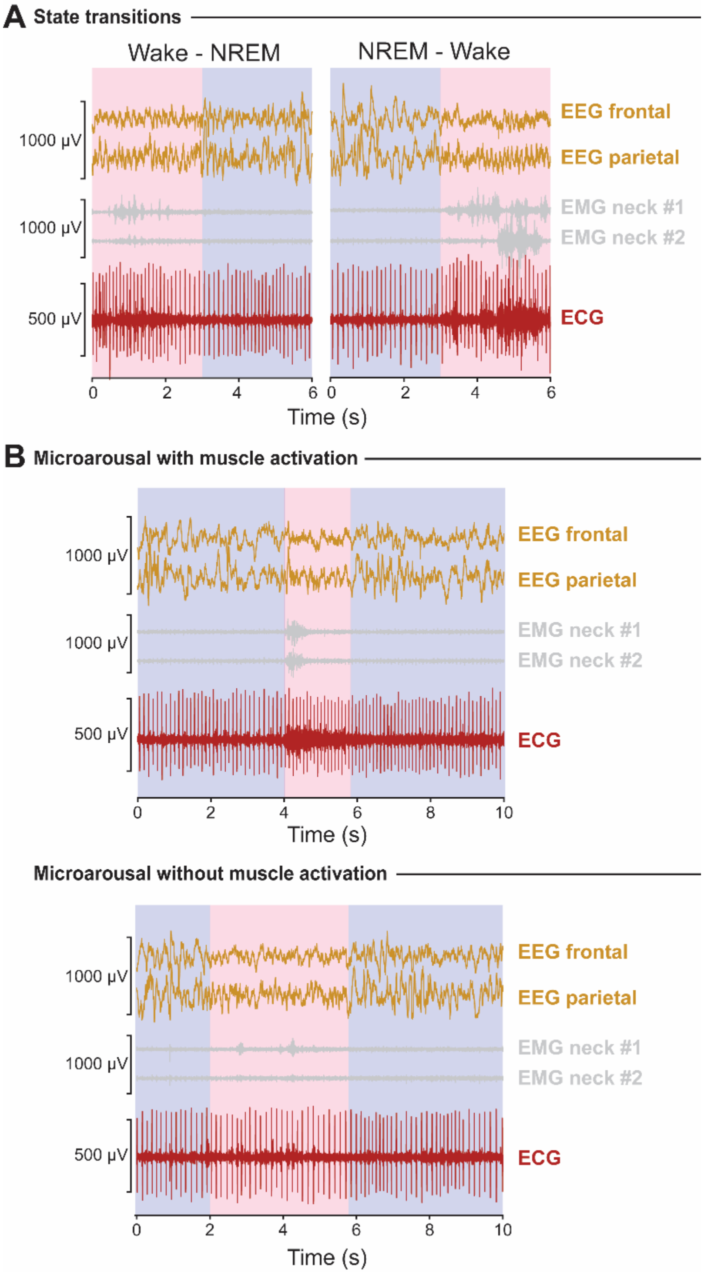

Supplementary Figure 1. Examples of state transitions. A) Example state transitions from wake to NREM (left) and from NREM to wake (right). **B)** Example microarousals during NREM with motor activation (top) and without motor activation (bottom). Magenta background represents wake and blue background represents NREM.

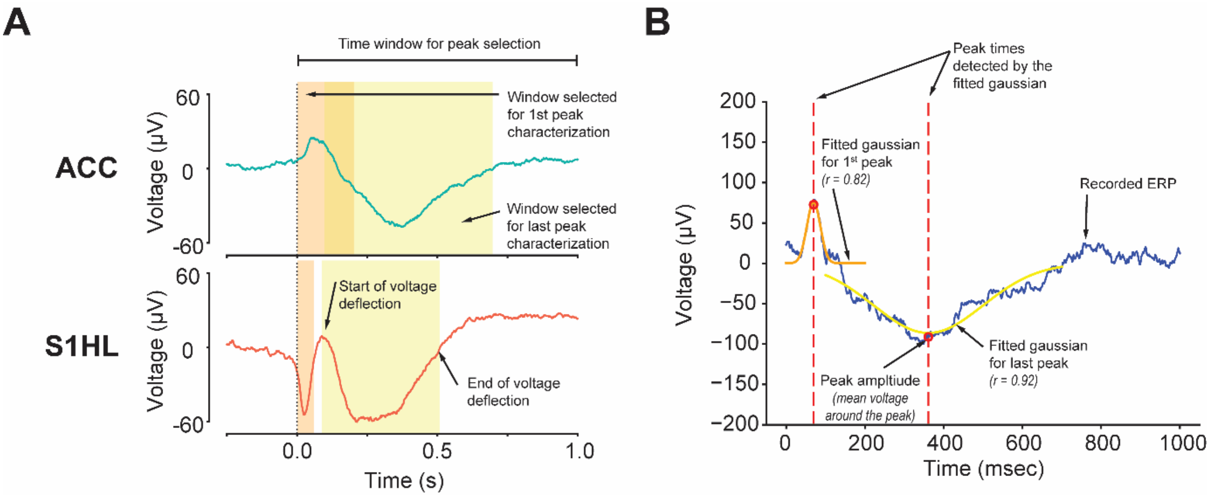

Supplementary Figure 2. Illustration of the collection of peak amplitude and times. A, Global SSEPs for ACC and S1HL illustrating the manually chosen boundaries for each peak and the definition of the boundaries based on the start and the end of the voltage deflection. **B,** Example SSEP from the ACC for one condition. Each peak of the Evoked Potential (blue line) was separately fitted to a gaussian (1st gaussian, orange; last gaussian, yellow). The peak of the fitted gaussian provided the time for each peak (dashed red lines). A Pearson's correlation was run for each peak to confirm the goodness of the fit between the Gaussian and the real data. The voltage average around the peak provided the peak amplitude (red dot).

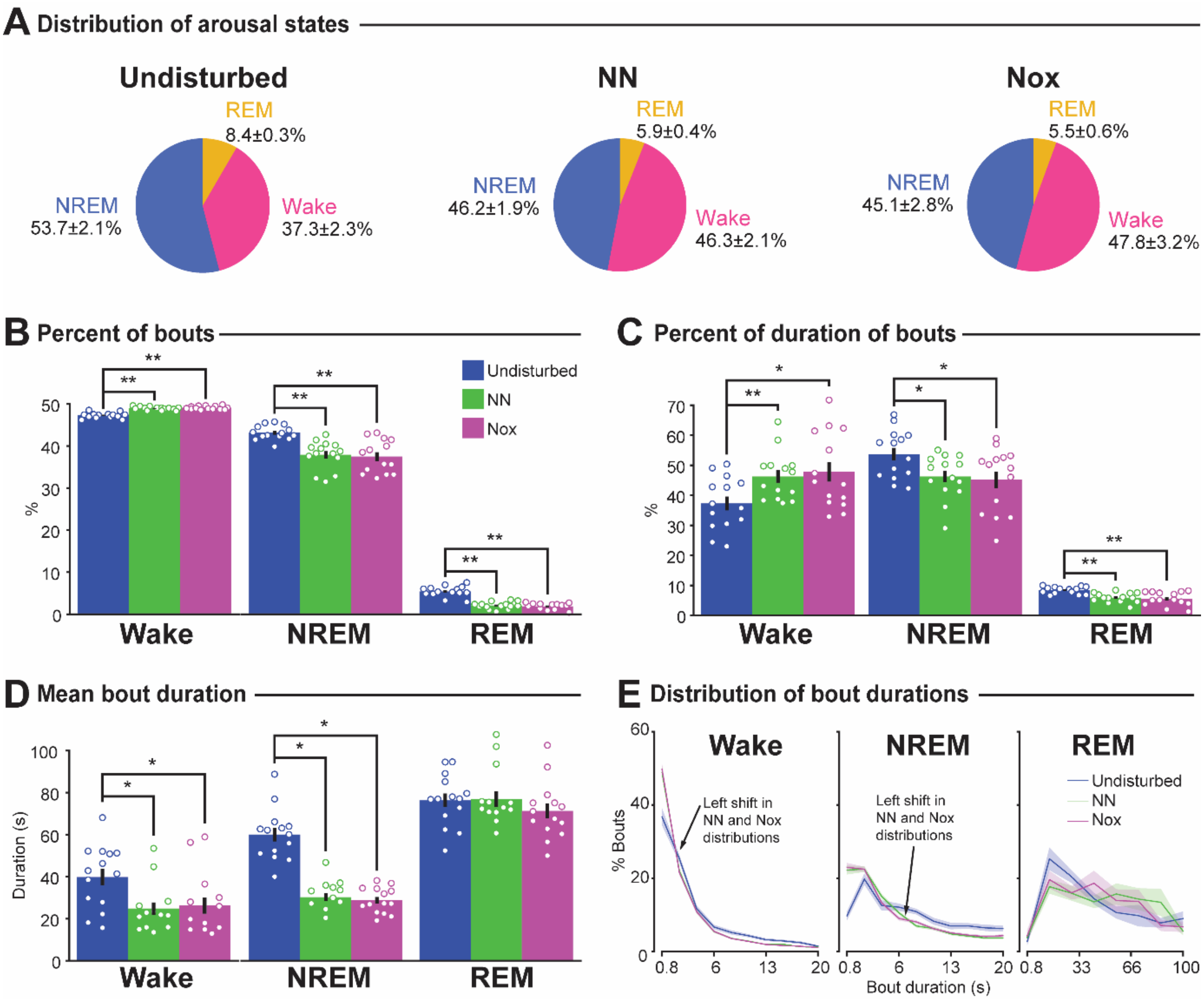

Supplementary Figure 3. Comparison of sleep characteristics in undisturbed sessions with NN and Nox sessions. A, Percentage of each arousal state. **B,** Percentage of the number of bouts recorded per session. **C,** Percentage of the duration of each bout of the total duration of each arousal state. **D,** Mean duration of each arousal state. **E,** Distribution of the duration of bouts. Data are represented as mean ± SEM. **B-C,** Each dot represents an animal. A t-test was run for each comparison. N=14. *, p<0.05; **, p<0.01.

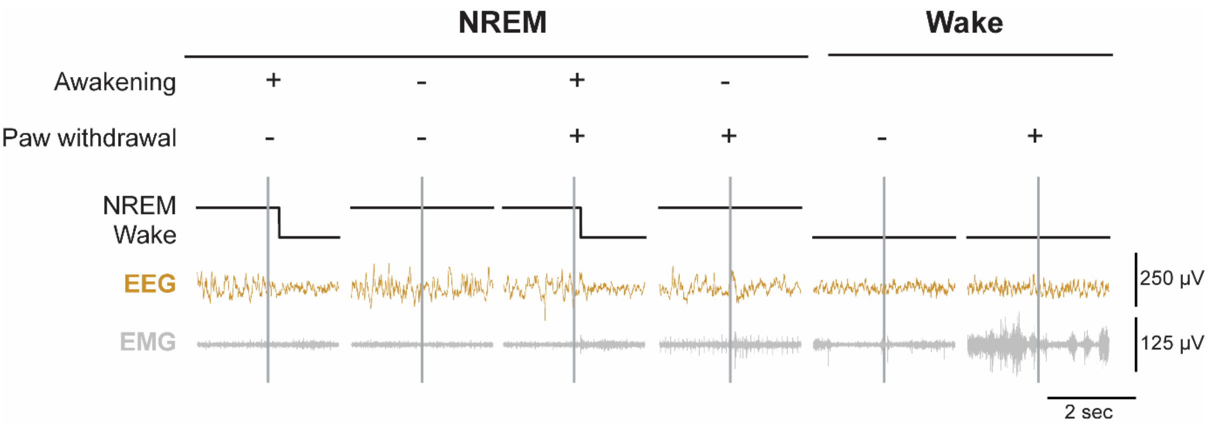

Supplementary Figure 4. Definition of behavioral responses. Example traces of the different behavioral responses of the recorded mice. Vertical lines represent the stimulation onset.

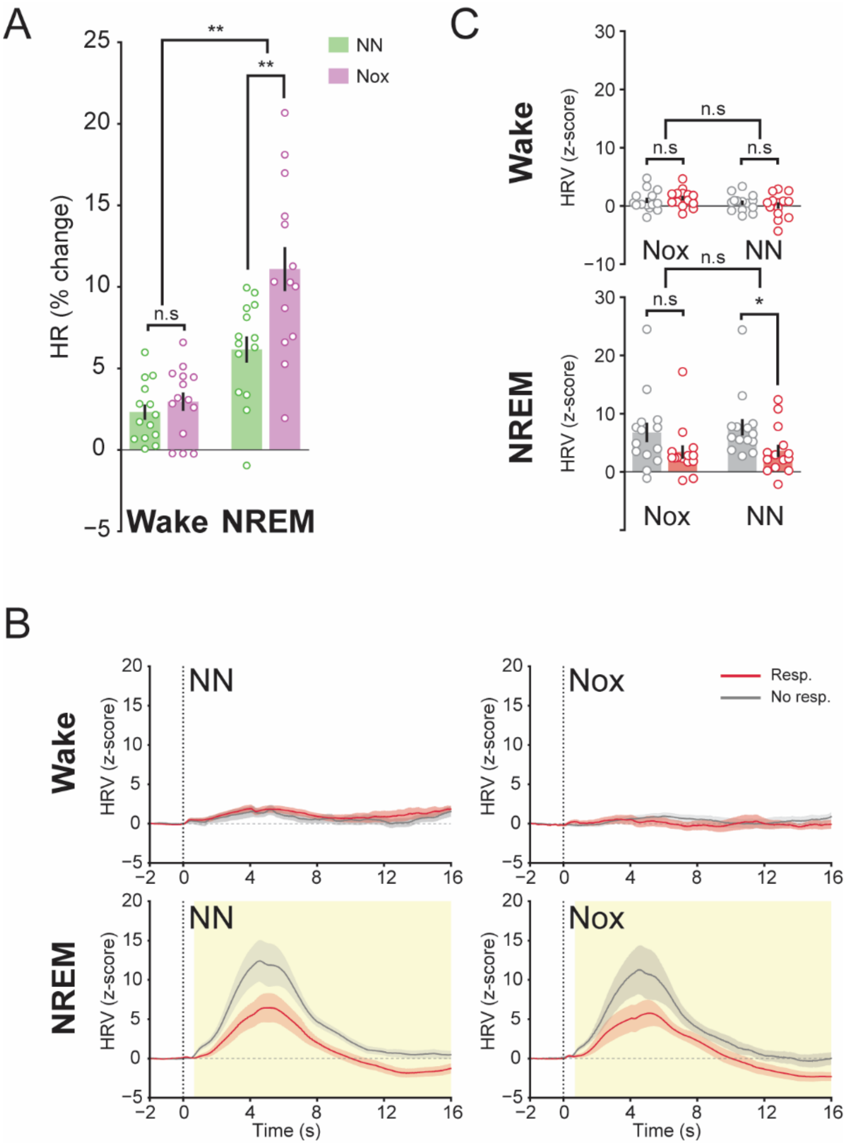

Supplementary Figure 5. Stimulus-evoked changes in heart rate variability. A, Heart rate variability (HRV) in z-score from baseline. **B,** Bar graphs of the mean HRV of an 8 seconds window between 2 and 10 seconds after stimulation onset. **C,** HRV time course as z-score. Yellow boxes represent p<0.05, FDR corrected. Each data point represents one animal (N=14). Data were represented as mean ± SEM. *, p < 0.05; **, p < 0.001; n.s., not significant. See Supplementary Tables 1 and 2 for ANOVA statistics.

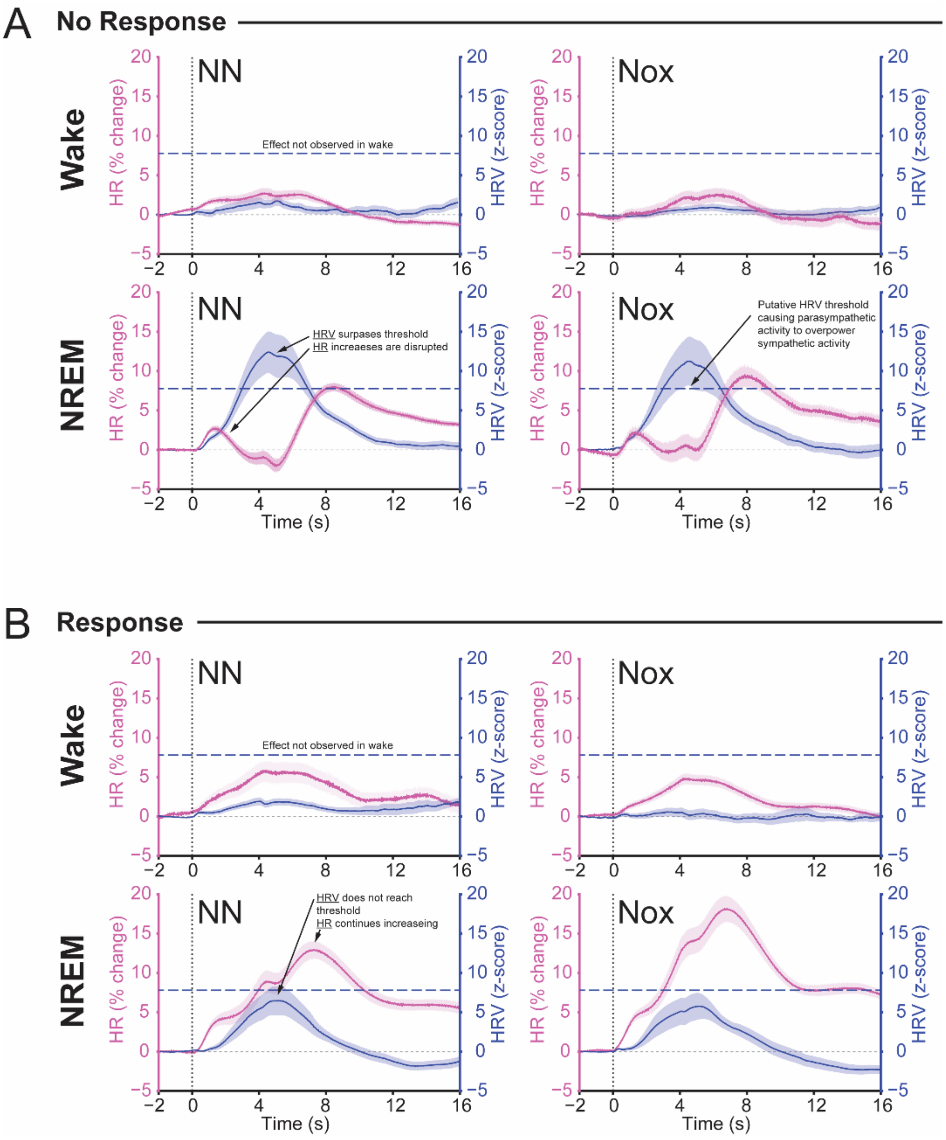

Supplementary Figure 6. Interactive effects between the heart rate and the heart rate variability. A, Stimuli that did not evoke overt behavioral responses. **B,** Stimuli that evoked overt behavioral responses. Data were represented as mean ± SEM. Vertical dotted line represents the stimulation onset. N=14.

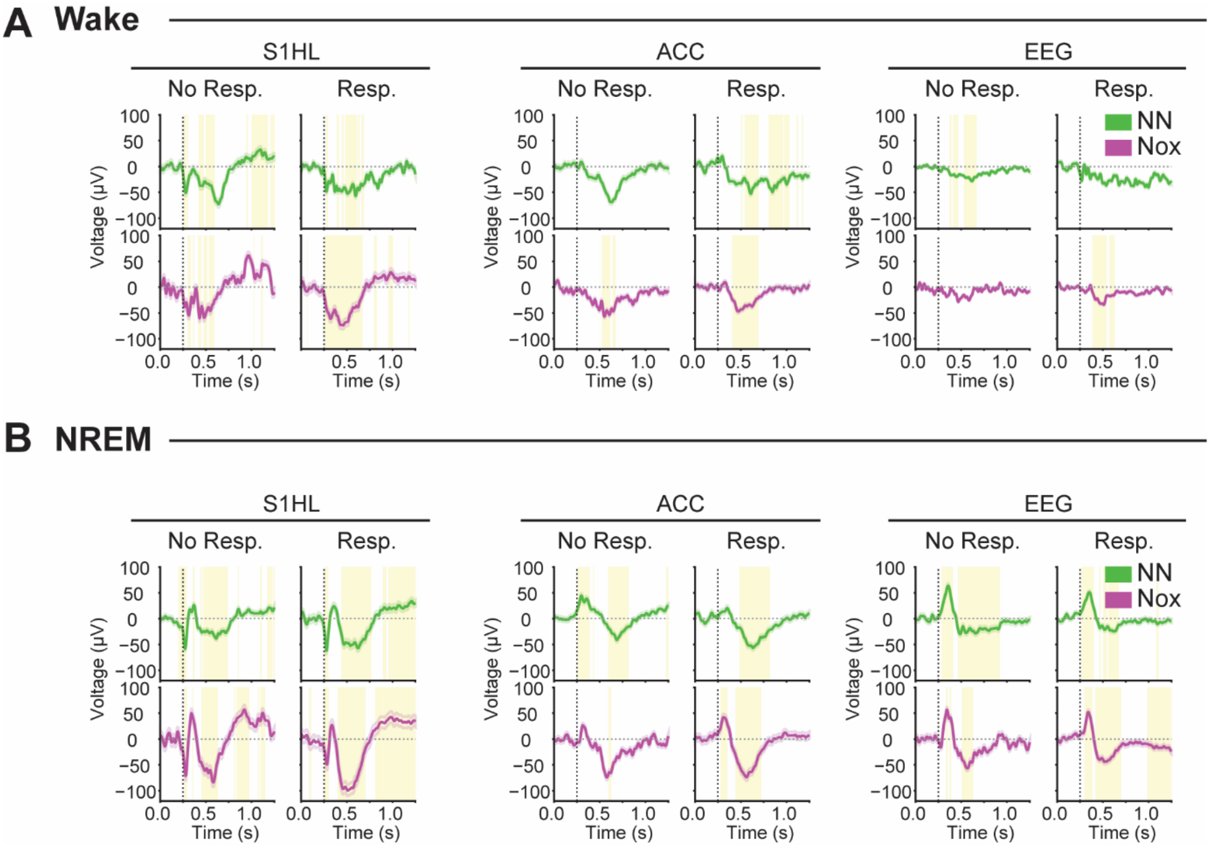

Supplementary Figure 7. Statistical confirmation of Somatosensory Evoked Potentials. **A,** SSEPs recorded in wake for each recording site, divided by type of stimulation and behavioral response. **B,** SSEPs recorded in wake for each recording site, divided by type of stimulation and behavioral response. Yellow boxes represent p<0.05, FDR corrected, indicating the signal is statistically different than zero. Data were represented as mean ± SEM. N=17. *, p < 0.05; **, p < 0.01. See Table 2 for statistics.

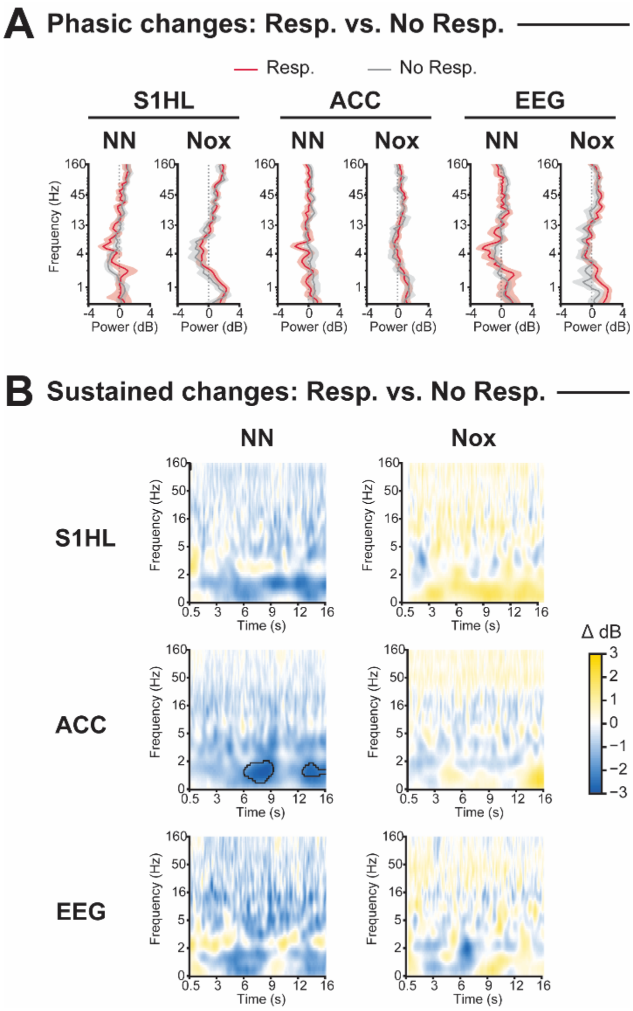

Supplementary Figure 8. Comparison of the spectral properties of stimuli with and without a behavioral response during wake. A, Spectral profiles of the phasic response (0 – 400 msec post-stimulation onset) in dB. Vertical dotted line at zero defines no change from baseline. Data were represented as mean ± SEM. Yellow shaded areas indicate statistically significant differences at p<0.05, FDR corrected. **B,** Differential sustained response. Black contour lines delineate statistically significant differences between response and no response (p<0.05, FDR corrected), N=17.

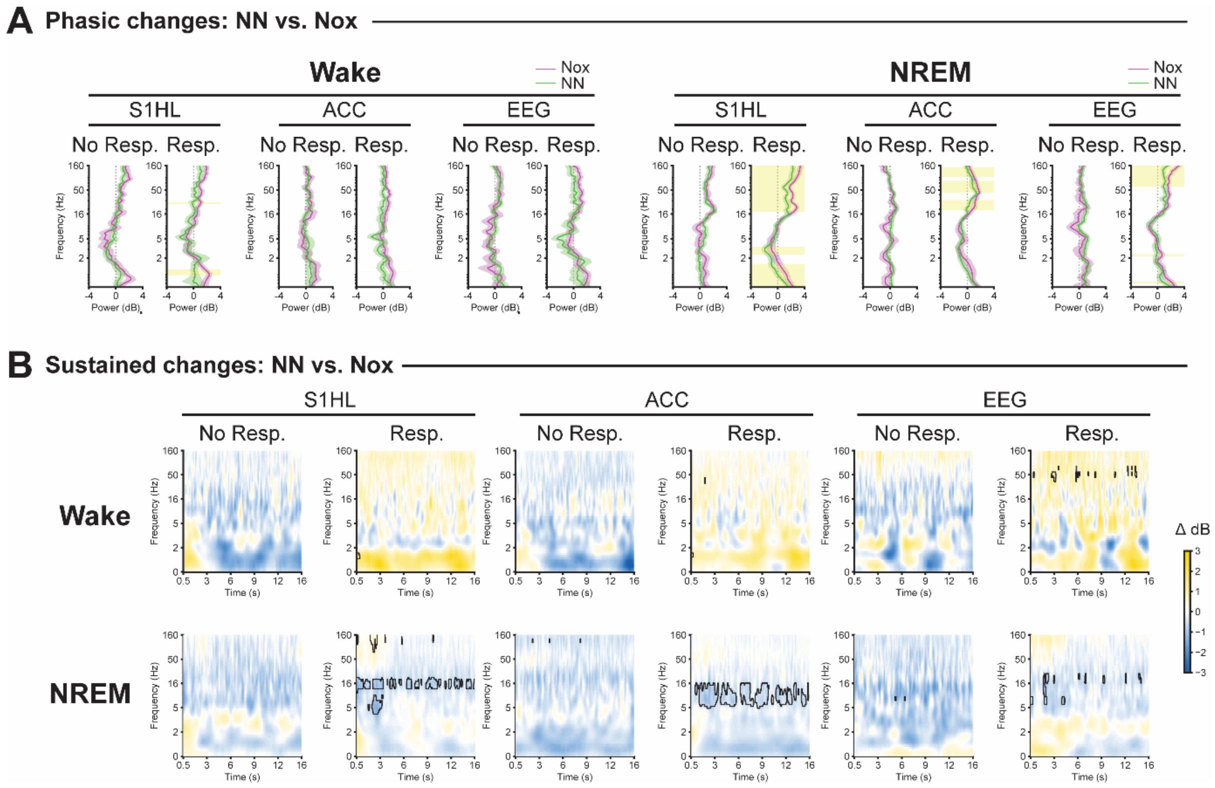

Supplementary Figure 9. Spectral properties comparing non-noxious and noxious stimulation. A, Spectral profiles of the phasic response (0 – 400 msec post-stimulation onset) in dB. Vertical dotted line at zero defines no change from baseline. Data are represented as mean ± SEM. Yellow shaded areas indicate statistically significant differences at p<0.05, FDR corrected. **B,** Differential sustained response resulting from the subtraction of non-noxious spectrograms from noxious spectrograms. Black contour lines delineate statistically significant differences between noxious and non-noxious (p<0.05, FDR corrected).
